# The Unusual Mesophases and Properties Exhibited by a Family of Glycosteroids

**DOI:** 10.1002/chem.202403678

**Published:** 2025-01-31

**Authors:** Fahima Ali‐Rachedi, Nuno M. Xavier, Xiaoyang Yue, Stéphane Chambert, Feng Liu, Laurence C. Abbott, John N. Moore, Xiangbing Zeng, Stephen J. Cowling, Yves Queneau, John W. Goodby

**Affiliations:** ^1^ INSA Lyon, Universite Claude Bernard Lyon 1 CNRS CPE-Lyon ICBMS UMR 5246 Bâtiment Lederer 1 Rue Victor Grignard F-69622 Villeurbanne France; ^2^ Faculty of Science and Technology Univ Souk Ahras 41000 Souk Ahras PB 1553 Algeria; ^3^ Laboratory of Sciences and Technology of Water and Environment LSTWE 41000 Souk Ahras Algeria; ^4^ Centro de Química Estrutural, Institute of Molecular Sciences Faculdade de Ciências Universidade de Lisboa Ed. C8, 5° Piso, Campo Grande 1749-016 Lisboa Portugal; ^5^ Department of Materials Science and Engineering University of Sheffield Mappin Street Sheffield S1 3JD UK; ^6^ Department of Chemistry The University of York York YO10 5DD UK; ^7^ Shaanxi International Research Center for Soft Matter State Key Laboratory for Mechanical Behaviour of Materials Xi'an Jiaotong University Xi An Shi Xi'an 710049 P. R. China; ^8^ present address: School of Chemical Engineering and Technology Hebei University of Technology Tianjin 300401 P. R. China

## Abstract

In this article we describe research on the synthesis and characterization of a family of “Janus” amphiphiles composed of disaccharide head groups and alkaloid units joined together via a methylene linker, and bearing a lateral aliphatic chain of varying length. The condensed phases formed by self‐organization of the products as a function of temperature were characterized by differential scanning calorimetry, thermal polarized light microscopy, and small angle X‐ray scattering, allied with computational modelling and simulations. Structural studies on heating specimens from the solid showed that some homologues exhibited lamellar, columnar and bicontinuous mesophases, whereas the same homologues revealed different phase sequences on cooling from the amorphous liquid. We explore these unusual results, which are revealed via supercooling.

## Graphical abstract

This article describes the synthesis and characterization of a family of “Janus” amphiphiles comprising disaccharide head groups and alkaloid units joined via a methylene linker, and bearing a lateral aliphatic chain. The condensed phases formed by self‐organization of the products as a function of temperature were characterized by DSC, thermal POM, and SAXS, allied with computational modelling and simulations.

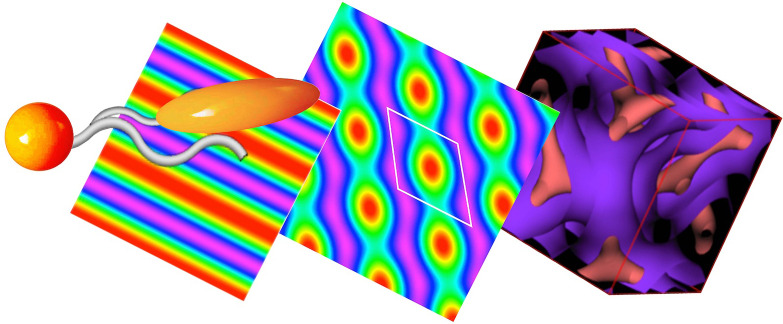

## 1 Introduction

One of the earliest reports on the liquid‐crystalline properties of materials derived from plants was made on α‐solanine. Solanine is a glycoalkaloid poison that is found in various plant species of the nightshade family, for example potato, tomato, and eggplant. It is generally known that potatoes exposed to light can turn green and become toxic to eat; this is because they produce solanine and chaconine naturally as defence mechanisms against pests, and in particular, potato leaves, stems, and shoots are naturally high in glycoalkaloids. α‐Solanine has a relatively rigid rod‐like dichotomous structure with one end being dominated by polar, H‐bonding hydroxy groups, and the other end by an alicyclic hydrocarbon with the potential to exhibit weak van der Waals (VDW) interactions. The dichotomy supports the formation of thermotropic liquid crystal phases,[^1^, ^2^] with the rigid steroidal and trisaccharide units inducing the transition temperatures to be high. As a consequence, α‐solanine has a melting point of 263 °C and a clearing point of 283 °C. However, there is little information on its lyotropic behaviour, and nor for structurally related materials.

Similar glycoalkaloids can be found in bacteria, some of which are relevant to humans. For example, *Helicobacter pylori* is a bacterium that can be found in the digestive tract of humans.^[3]^ It can be dangerous as it can cause ulcers to form in the lining of the stomach or small intestine. It is thought that such infections potentially support the formation of stomach cancers. α‐CAG is a 6‐*O*‐tetradecanoyl glycosteroid that has been isolated from *Helicobacter pylori*. Like α‐solanine, it has a structure based on a steroidal moiety linked to a sugar unit, but in addition it also possesses a C_13_ terminal aliphatic chain, thereby making the structure trichotomous. This means that the material will be likely to exhibit thermotropic liquid crystal behaviour, but be much less likely to be lyotropic or exhibit amphitropic tendency.

**Figure 1:**
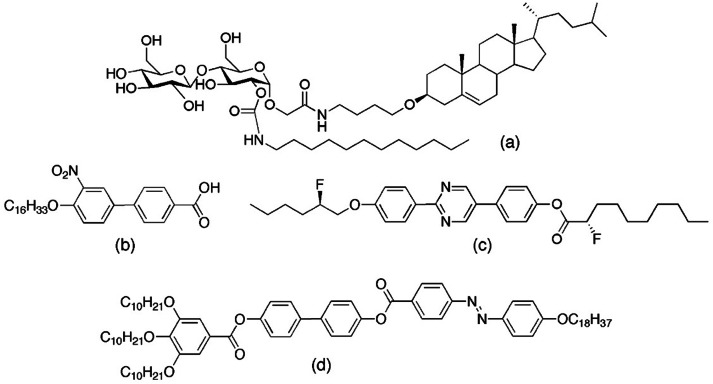
Four compounds that exhibit different phase behaviours that are not necessarily reversible on heating and cooling cycles from the solid state to the amorphous liquid.

To investigate the amphiphilicity of synthetic materials based on natural products, across a homologous series of compounds, we synthesised compounds with sugar and steroidal moieties bound together as in the case of solanine. One particular material, *N*‐{4‐[(3β)‐cholest‐5‐en‐3‐yloxy]butyl}‐2‐({2‐*O*‐[(dodecylamino)carbonyl]‐4‐*O‐*β‐D‐glucopyranosyl‐α‐D‐glucopyranosyl}oxy)acetamide, structure (a) in Figure 1 was shown^[4]^ to exhibit remarkable melting and cooling behaviour. On heating it forms a cubic, bicontinuous mesophase, but on cooling from the liquid this is replaced by the formation of a lamellar phase. Normally on heating and cooling for thermotropic liquid crystals the phase sequences are reproducible, thus for this material it should have exhibited cubic phases on heating and cooling.

All but a handful of thermotropic liquid crystals show cyclical thermal phase behaviour, those few that do not possess H‐bonding, chiral moieties, or polycatanar structures, as shown in Figure 1 for compounds (b to d), thereby demonstrating that such phase behaviour covers compounds possessing a relatively wide variety of molecular motifs.

In Figure 1, 4′‐hexadecyloxy‐3′‐nitrobiphenyl‐4‐carboxylic acid (b), has been shown to exhibit two lamellar and one cubic phase on heating, but on cooling a columnar modification can replace the cubic phase, and without mesophase reversibility occurring on heating again.[^5^, ^6^, ^7^, ^8^] Chiral compound 2‐{4‐[(*R*)‐2‐fluorohexyloxy]phenyl}‐5‐{4‐[(*S*)‐2‐fluoro‐2‐methyldecanoyloxy] phenyl}pyrimidine (c), has been found to exhibit lamellar and cubic phases on heating, but with a cubic phase below the lamellar phase on cooling, and in some circumstances it can exhibit two different phases at the same temperature.^[9.10]^ The azo‐polycatenar compound (d) has similar phase behaviour to (a) in that it possesses a cubic phase which appears only the heating cycle.^[11]^ Comparing compounds (a) to (d) they have little in common with respect to their chemical structures, and only compound (b) has a homologue with respect to the aliphatic chain length, i. e., C_18_H_37_ versus C_16_H_33_. Surprisingly, the results for the mesomorphic behaviour of the molecular materials extends from 1981[^12^, ^13^] to the current day, demonstrating the rarity of such phase behaviour, but they also indicate the broadness of the chemical architectures with no one material being of the same or similar family.

**Figure 2:**
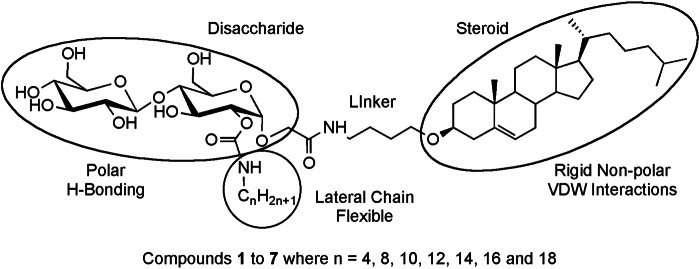
General structure of the target glycosteroids, where the lateral aliphatic chain was varied in length of a function of (n).

Consequently, our research programme was split into two objectives. The first was to examine the melting and cooling behaviour plus the phase transitions across a homologous series of materials. The series of materials targeted for synthesis was based upon compound (a) as shown by the generalized structure in Figure 2. The second was to investigate the producibility and reversibility of the phase transitions with the techniques of analysis, i. e., polarized light microscopy (POM), calorimetry (DSC), and X‐ray diffraction (SAXS), and to examine molecular self‐organization through computer simulations.

The three circled motifs of the trichotomous structure shown in the figure are as follows: (i) a polar, hydrogen‐bonding and chiral disaccharide, (ii) a chiral non‐polar rigid hydrocarbon steroid, and (iii) a laterally appended flexible non‐polar aliphatic chain. The disaccharide and the steroidal units are similar in size, with a butylene linkage joining them together, therefore the combined “Janus” architecture is consistent throughout our studies. The one variation in the structure is the length of the lateral aliphatic chain (C_n_H_2n+1_). When it is short it is buried in the central linking section, and as its length is increased it protrudes beyond the steroidal unit, thereby changing the hydrophilic/hydrophobic balance within the family of materials.^[14]^

## 2 Syntheses of Materials

**Figure 3:**
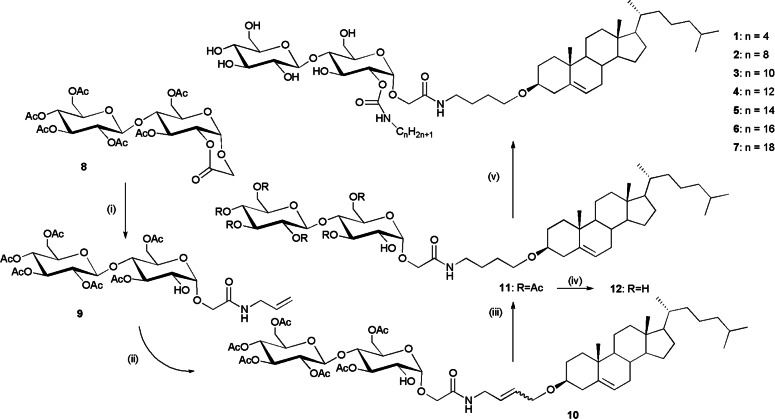
Synthesis of the target materials. Reagents and conditions: (i) allylamine, CH_2_Cl_2_, 24 h, RT, 96 %; (ii) Allyl cholesteryl ether, 5 % Grubbs‐Hoveyda II, CH_2_Cl_2_, 24 h, RT, 63 %; (iii) H_2_, Pd/C, THF, 1 h, RT, 95 %; (iv) MeOH/NEt_3_/H_2_O (8/1/1, v/v), 3 h, 40 °C, 92 %; (v) 1) alkyl isocyanate, DBU, CH_2_Cl_2_, 3 days, RT, 2) MeOH/NEt_3_/H_2_O (8/1/1, v/v), 3 h, 40 °C, 50–65 % from **11**.

The materials targeted were synthesized using the pathway shown in Figure 3, details for which are given in the Experimental Section and Supplementary Information (SI). The scheme uses a similar strategy to the one developed for the synthesis of the corresponding monosaccharide compounds,[^14^, ^15^] and optimization of reaction conditions for the application to the disaccharide carbohydrate lactone **8** as the starting point.^[16]^ The key steps in this scheme are the addition of allylamine to the lactone **8** giving the allylamide **9**, and the olefin cross‐metathesis with allyl cholesteryl ether^[17]^ as the connecting step leading to the glycosteroid **10** having an unsaturated butenyl spacer between the carbohydrate and the steroid moieties. Selective hydrogenation of this butenyl spacer gives intermediate **11**. This material can be either directly deacetylated to give the unprotected cellobiose adduct **12**, or, when possessing a single hydroxy function at position 2, transformed in a two‐step procedure including first the reaction of OH‐2 with alkyl isocyanates of different chain lengths, and subsequently the deprotection of the acetyl groups to afford the targeted amphiphilic urethanes **1**–**7** having the carbamoyl group at O‐2.

## 3 Results and Analysis

We turn now to the characterization of the mesophases exhibited by the homologues and the evaluation of the various phase transitions using POM, DSC, X‐ray diffraction, and computer simulations, for which the details of the techniques can be found in the Experimental Section. And for each of the studies we begin with the investigation of compound **6**, because it has two lipid chains of similar length, which is important when considering the hydrophobic/hydrophilic balance.

**Figure 4:**
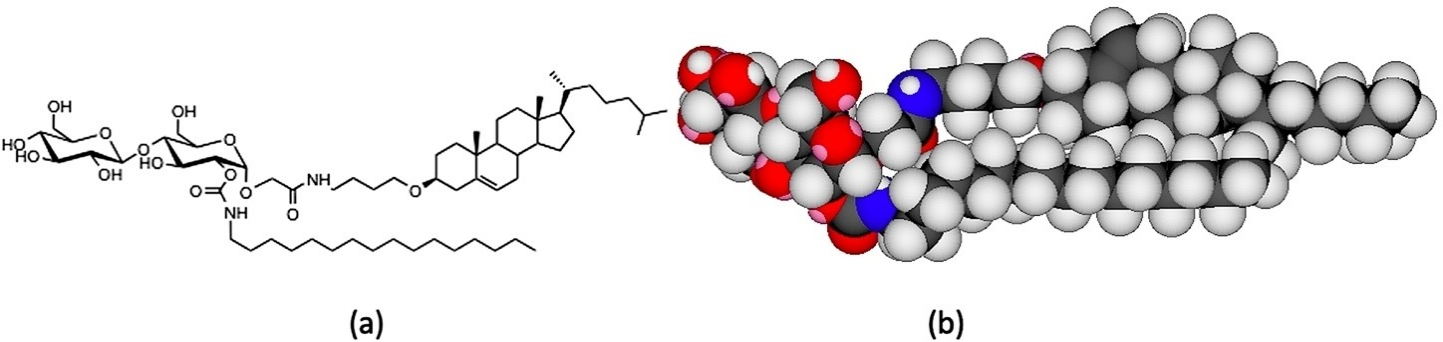
(a) The chemical structure of compound (**6**), and its space filling form determined by ChemDraw 3D minimised using the MM2 energy minimisation toolkit.

For the analysis of the structures of the mesophases it is also important for to have details of the molecular dimensions. For compound **6**, Figure 4(a) shows its chemical structure in its most elongated form, whereas in Figure 4(b) it is shown in its space‐filling form determined by ChemDraw 3D for a single molecule. The length of the hexadecanyl chain was determined to be approximately 2.36 nm (23.6 Å), the steroidal bearing moiety was ~2.46 nm (24.6 Å), and the disaccharide moiety ~1.02 nm (10.2 Å). In the simulated model, in its intramolecular H‐bonded form, the overall molecular length was found to be approximately ~3.42 nm (34.2 Å). It should also be borne in mind that such space filling structures for molecules in liquid crystals will, in general, possess rapid conformational and dynamic motion as shown in various neutron and X‐ray scattering studies, and computer simulations,[^18^, ^19^] and that predictions about which potential mesophase structure might be formed may be determined from gross features such as hydrocarbon volume, the area of the head group, and the critical length of a hydrocarbon moiety.[^20^, ^21^, ^22^]

### 3.1 Thermal Polarized Light Microscopy Studies

Utilizing POM to determine mesophase identity and transition temperatures as described in the Experimental Section,^[23]^ starting with compound **6**, on heating a pristine sample at a rate of 5 to 1 °C min^−1^, the material melted at 110 °C. Subsequent heating ultimately resulted in a transition to isotropic liquid at a temperature of 230 °C.

**Figure 5:**
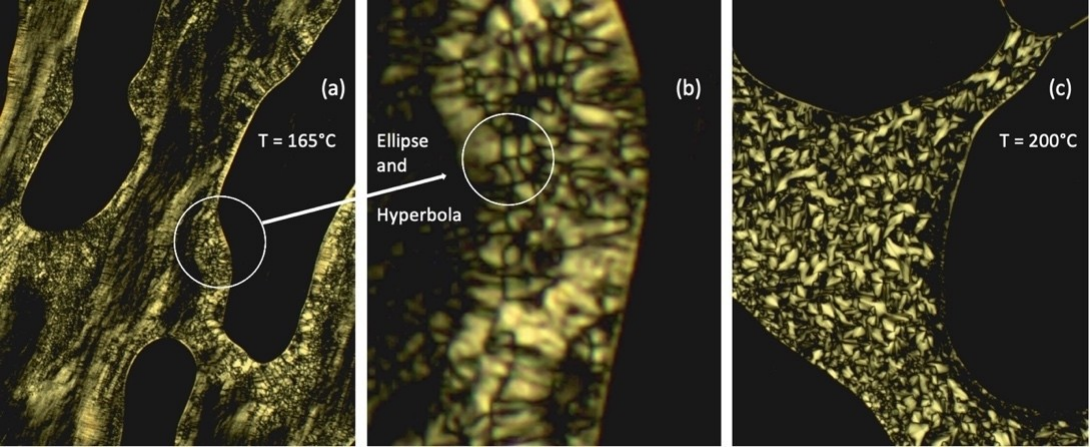
Photomicrograph under crossed polarizers (x100) for compound **6**, (a) heating at 165 °C, (b) expansion of (a) showing focal‐conic defects with elliptical and hyperbolic lines of optical discontinuity (circled), and the defect texture of (c) (x100) shown on cooling from the isotropic liquid at a temperature of 200 °C.

Using thermal microscopy, under crossed polarizers, on heating **6** exhibited a birefringent phase, as shown in Figure 5, at a temperature of 165 °C. Examination of the defect textures generated by the mesophase suggests they could be formed by either a smectic or columnar phase. Closer examination of the portion of the texture in the white circle in Figure 5(a) shows that the texture in the left side of the circle is homeotropic and therefore the phase is optically uniaxial. Towards the centre of the white circle in Figure 5(b), elliptical and hyperbolic lines of optical discontinuity are present indicating the presence of focal‐conic Dupin‐cyclides associated with a lamellar phase.[^24^, ^25^] Along with the closer examination of sections of the defect textures, Figure 5(a) also shows textures associated with shear flow patterns, indicating that the mesophase has a relatively low viscosity. The bright and dark regions in the flow patterns indicate homeotropic and homogenous alignment. These combined observations confirm that the mesophase is smectic A.

Cooling from the isotropic liquid, the mesophase formed is shown in Figure 5(c) at a temperature of 200 °C. Along the edge of the right‐hand air bubble there are black regions bound to the outer edge of the mesophase, along with focal‐conic domains. These textures also indicate that the mesophase is optically isotropic and smectic A. At temperatures between 165 and 200 °C, partially birefringent and homeotropic flow textures were observed, but with no distinct isotropic regions being present. In comparison however, it should be noted that flow birefringence has also been reported previously for the cubic phase of 4′‐octadecyloxy‐3′‐biphenyl‐4‐carboxylic acid by Winsor.^[26]^

**Figure 6:**
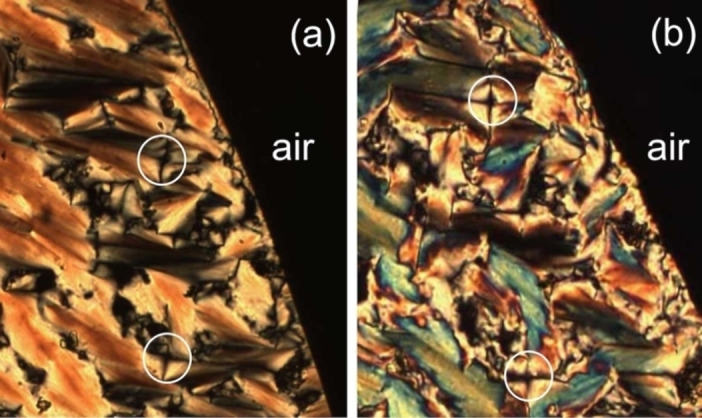
Defect textures (x100) formed by compound **6** under crossed polarizers in transmission optical microscopy. (a) The focal‐conic texture (an ellipse and hyperbola are circled) obtained upon cooling at 171 °C; (b) the same area, still exhibiting focal‐conic defects (circled) at room temperature.

In order to investigate the phase sequence observed for the initial cooling of compound **6**, we heated a specimen to a temperature just below the isotropization point. The material was then allowed to form a focal‐conic texture, as shown in Figure 6(a). On cooling, the phase became increasingly stabilized and remained in place right through the temperature range down to room temperature whereupon glassification occurred, as shown by the comparative textures as indicated by the white circles in Figure 6(a) at 171 °C and Figure 6(b) at room temperature. Over several days at room temperature, the glassified liquid crystal phase was retained in its defect textural form. Mechanical shearing of this defect texture was difficult to achieve indicating that the mesophase had formed a glass‐like solid. Comparative DSC showed potential solidification at approximately 75 °C. Overall, the results show that the mesophase of compound **6** is a type of smectic A phase,^[27]^ with the only other possibility being hexatic B.^[28]^ It appears it is not columnar which normally does not exhibit focal‐conic defects. However, the birefringence seen in mid‐temperature range indicates that cubic phase formation is also a possibility, but which was not supported via calorimetry.

**Figure 7:**
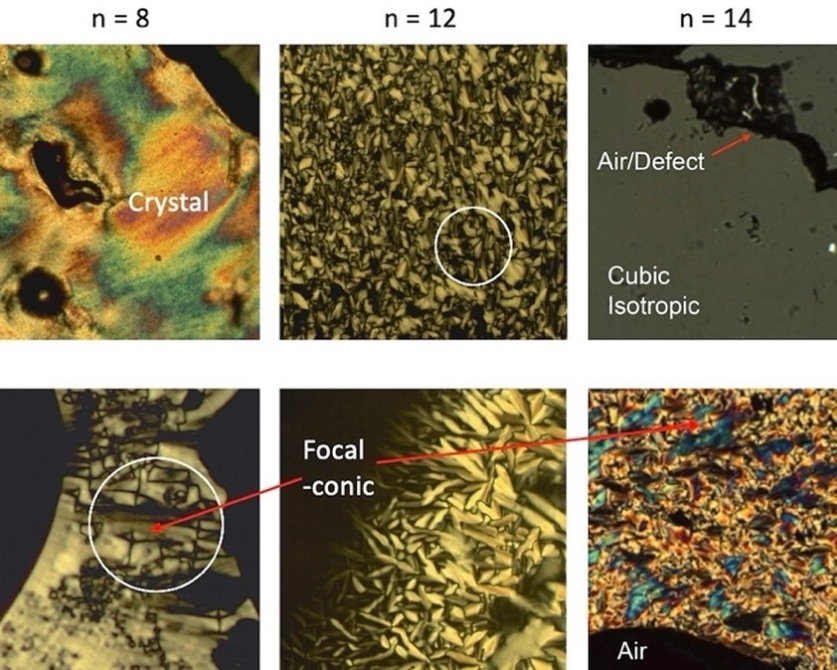
The defect textures of compounds **2** (n=8), **4** (n=12) and **5** (n=14) at a magnification of x100.

We now turn to the other homologues of the family of glycosteroids (compounds **1** to **5**, and compound **7**) and examine their transition temperatures determined by POM, as shown in Table 1. All of the materials appeared to exhibit smectic A phases as characterized from their focal‐conic and homeotropic defect textures.[^24^, ^25^] Compounds **3**, **4**, and **5** also exhibit cubic phases as seen from their viscous, isotropic textures. Compound **5** also appears to possess a columnar phase near to the clearing point, and at a lower temperature an unidentified X phase by microscopy. The defect textures of compounds **2**, **4**, and **5** are shown together in Figure 7.

**Table 1:** The transition temperatures (°C) determined by thermal optical microscopy for compounds **1** to **7** and the unsubstituted parent compound **12**.^[a]^ The melting points are given from the solid (Cr) to the mesophase using DSC.

Cpd	n	Transition Temperatures (°C) on Heating^[a]^	Transition Temperatures (°C) on Cooling
**12**	0	Cr 168 SmA 268 Iso Liq (decomposition)	
**1**	4	Cr 150 SmA 290 Iso Liq (decomposition)	
**2**	8	Cr 125 SmA 222 Iso Liq	
**3**	10	Cr 120 SmA 169 Cubic 186 SmA 220 Iso Liq	
**4**	12	Cr 80–100 Cubic 220 SmA (Col) 230 Iso Liq	Iso Liq 230 SmA then down to glass at RT
**5**	14	Cr 120 X 153.6 Cubic 180 SmA (Col) 215.5 Iso Liq	Iso Liq 205 SmA then down to glass at RT
**6**	16	Cr 110 SmA 230 Iso Liq	
**7**	18	Cr 150 SmA 240 Iso Liq	

For compound **2** (n=8) two textures are shown in Figure 7. The lower texture shows focal‐conic domains (red arrow to the white circle) interspersed with black areas (to the right), which are homeotropic where the optic axis is pointing out of the page, and the optically extinct nature of the texture indicates that the phase is optically uniaxial. The upper texture is a pattern exhibited by the crystal phase. These two textures confirm that there is one mesophase that can be classified as smectic A, (SmA or lamellar).

Compound **4** (n=12) also exhibits two textures as shown in Figure 7. The upper photomicrograph, taken at 195 °C shows a texture composed of fans which do not appear to have ellipses or hyperbolae connected to Dupin cyclides. However, there is one defect (circled) that has lines of optical discontinuity, which confirms that the phase is lamellar, and smectic A. The lower photograph shows the transition from the liquid phase to the smectic A phase on cooling. Again, the birefringent textures showing fan‐like defects do not appear to exhibit lines of optical discontinuity, and as a consequence the defect pattern appears to support the presence of a columnar phase. On further cooling to 170 °C, parts of the texture that possessed optically extinct domains experienced a phase transition where the edges of the domain sharpened to give a well‐ordered phase that had isotropic optical properties, and hence is cubic. Therefore, on heating compound **4** appears to have a transition from the solid state to a cubic mesophase, which then transforms into a lamellar phase that may also share phases with a columnar phase. Upon cooling from the isotropic liquid there is a transition from the liquid to the lamellar phase which exists down to a temperature below that which exhibited a cubic phase. Thus, the cyclic sequence of phases for compound **4** is not reversible with respect to heat and cooling.

The third pair of photomicrographs exhibited in Figure 7 for compound **5** (n=14) shows two very different textures. The lower picture shows focal‐conic domains of the smectic A phase, where optical discontinuities in the fan‐like domains are indicated by the red arrow. The discontinuities appear as black crosses of curved lines, which are associated with ellipses and hyperbolas of Dupin cyclides.[^24^, ^25^] The upper picture is the optically extinct texture of an isotropic phase, where the polarizers of the microscope have been slightly uncrossed so that the texture appears grey. This phase is clearly not liquid–like as shown by the nature of the dark defect in the top‐right of the texture. Moreover, mechanical shearing of the phase does not induce any bright defects indicating that the phase is isotropic and viscous, and therefore cubic. The textures exhibited by compound **7** are not shown in Figure 7 because they are very similar to those shown in Figure 5 for compound **6**.

### 3.2 Calorimetry Studies

**Figure 8:**
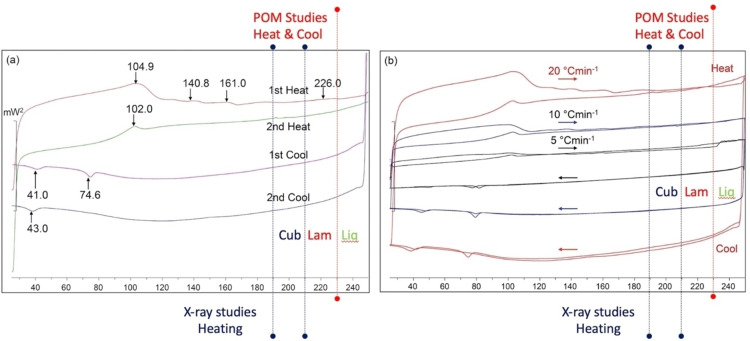
Differential scanning calorimetric thermograms (mW^2^/°C) for compound **6**, (a) shows first and second heating and cooling traces taken at rates of 20 °C min^−1^; (b) Shows scans taken at 20, 10 and 5 °C min^−1^ on the first and second heating and cooling cycles.

As with the earlier sections we start with the investigation of the thermograms of compound **6**. The first DSC trace shown in Figure 8(a) displays the first and second heating and cooling traces taken at scan rates of 20 °C min^−1^. The first heat shows some fluctuations above the melting point of 104.9 °C, at 140.8 and 161.0 °C respectively. However, they were not reproduced in the second heating cycle, indicating they are not associated with melting or phase transitions, but are artifacts probably associated with pan and/or lid movement. The baselines of the two scans are very similar if the broad peaks are omitted, which indicates that there is little or no decomposition at or after the phase transition to the liquid. For all of the thermograms the transition peaks appear to be broad, and in some cases very small, and so they were evaluated via the second order differentiation of each scan. An important observation to consider with each peak was the small change in the heat capacity across the transition, which indicates that transitions are not second order as one would observe a step in the baseline. As the enthalpies of the transitions are small, the mesophases either side of a peak potentially have similar structures and properties with respect to periodic and dynamic ordering.

Conversely, for compound **6**, the first and second cooling cycles exhibited some differences in the processes occurring towards recrystallisation. The first cooling cycle shows two transitions at 74.6 and 41.0 °C. These occur below the melting point and are either phases produced monotropically or they are two crystal forms created upon solidification. POM did not detect mesophase formation at these temperatures, and therefore we assume the peaks are two different crystal forms. For the second cooling cycle only the lower temperature solid phase was observed, with a peak maximum of approximately 43 °C. This temperature for the second cooling cycle was similar to that for the lower temperature peak of the first cool. The peak shapes for the two are similar, which suggests the peak at 43 °C is for the formation of the same crystal modification as seen in the first cooling cycle. As the three peaks associated with solidification are above room temperature, it suggests that **6** was also in its solid form when the microscopy studies, reported previously, were made at room temperature.

The second set of thermograms for compound **6** is shown in Figure 8(b) for scans rates of 20, 10 and 5 °C min^−1^ on first and second heating and cooling cycles. These studies show that kinetic behaviour has an effect on the phase transitions with the faster scan rates showing reproducible behaviour for the solidification process (~80 °C), whereas the slower scan shows that the lower temperature solid forms at ~40 °C. The first heating scan of 5 °C min^−1^ differs from the faster scans in that there is a step in the baseline at the transition to the liquid. An expansion of this trace shows that where the baseline smoothly approaches the transition, fluctuations due to pan settling occur in the liquid state once the transition was over.

The DSC thermograms show consistency, which is important when examining the baseline behaviour. For polarized light microscopy a lamellar phase was found to be present on heating to, and cooling from, the liquid phase, and in Figure 8 a red dotted line in both traces locates the phase transition. The first heating cycle for (a) and (b) shows a slight change in the baseline to mark the transition. Subsequent heats and cools show no change in the baseline.

Conversely, the X‐ray scattering studies (see below) show that a cubic phase is present along with the lamellar phase. The temperature range for the cubic phase is bounded by the two blue dotted lines in Figures 8(a and b). Again, an examination of the baseline shows no associated enthalpies for a phase transition between the dotted lines, except for some noise found in the first heating cycle in both sets of traces.

We find that there are no peaks in the DSC traces at the temperatures that phase transitions are found to occur in POM or X‐ray diffraction studies (see below). And yet, the transitions are consistent depending on which experimental study is being performed; for POM, only a lamellar phase is observed, whereas for X‐ray scattering, lamellar and cubic phases are always observed, indicating that the difference in the phase sequences is not due to artefacts. Alternatively, it might be that the expected transitions are so weak that any peaks associated with enthalpies cannot be detected, which means again that either side of the transition the phases are very similar in structure and properties, as noted above.

Lastly, we note from Figure 8, upon cooling from the liquid phase back into the liquid crystal state there are changes in the baselines, which possibly indicates that there is molecular organization occurring within the sample, which might be associated with cybotactic groups, as the transition is approached.

We conclude that, although the thermograms do not appear as clean as might be expected for classical liquid crystal materials, see SI Figures S1 and S2, the reproducibility with respect to sample origin and the slight variations due to kinetic processes, suggests that the poor reversibility of cooling and heating cycles in relation to the baseline indicates the results are real and without artefacts or due to impurities, and that the phase behaviours differ in the direction of temperature travel or possibility of monotropic behaviour.

All of the other homologues were investigated in the same manner as compound **6**, with only homologues **4** and **5** having differences in mesophase sequences on heating versus cooling. These sequences were not detected by DSC, but were found by POM and X‐ray diffraction.

### 3.3 X‐Ray Scattering Studies

Small angle X‐ray scattering experiments were performed on heating with data being taken at 10 °C intervals. The specimens were not heated into the liquid state because of the possibility of decomposition. In the studies, three liquid‐crystalline phases were found; the first and the lowest temperature phase consisted of modulated layers with a 2‐D lattice and *p*2 plane group symmetry; the second phase was cubic with the space group $Ia\bar{3}d$ and commonly known as a gyroid phase; and the third and highest temperature phase was a lamellar phase sometimes classified by smectic notation as SmA, and which agreed with the characterization made by POM.

**Figure 9:**
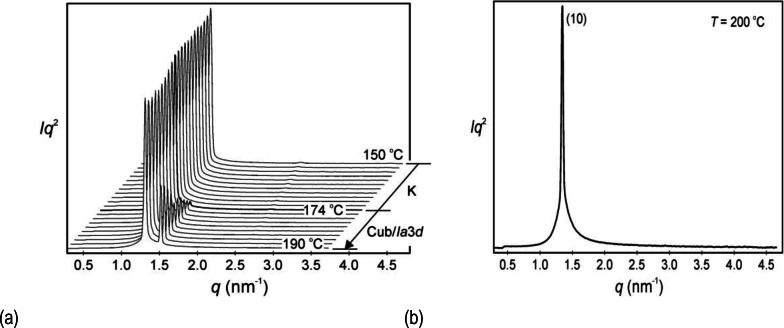
(a) X‐ray scattering profiles as a function of temperature (°C) for compound **6**. (b) Powder diffraction pattern for the lamellar phase recorded at 200 °C (intensities are in arbitrary units).

Following on from the POM and DSC studies for **6**, we start with its SAXS investigations, so that comparisons can be made with the previous microscopy investigations. Thermal microscopy of **6** showed that a lamellar phase existed between the liquid and the solid, and remained present until solidification; however, X‐ray scattering studies showed there was also the formation of an intermediate cubic phase upon heating in the temperature window from 174 to 190 °C, as shown in Figures 9(a and b). In these studies we also note it might be possible for the cubic phase to be monotropic for compound **6**, which might explain the discrepancy between results obtained from X‐ray scattering and POM studies.

Heating compound **6** to a temperature above 190 °C caused a phase transition from the cubic phase to the lamellar phase (SmA by POM). The SAXS X‐ray scattering pattern shows only one (10) reflection confirming a layered structure with a spacing of *d*=4.67 nm, as shown in Figure 9(b) at a temperature of 200 °C. The experimental and calculated *d*‐spacings of the observed SAXS reflections at 200 °C are given Table S1 in the SI where the intensity values are Lorentz and multiplicity corrected.

**Figure 10:**
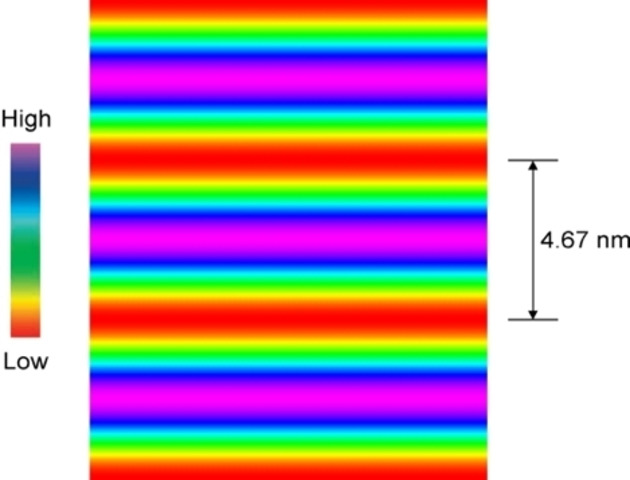
Reconstructed electron density map of the lamellar phase of compound **6** obtained from the powder pattern at 200 °C.

A reconstructed electron density map of the lamellar phase obtained from the powder pattern at 200 °C was produced as shown in Figure 10, with the coloured bars showing the high to low density profiles across the 4.67 layer spacing for the lamellar phase.

In comparison to the layer spacing of 4.67 nm, compound **6** was calculated to have a molecular length of approximately 3.42 nm, a difference of 1.25 nm. Therefore, the length of a molecule is too short to bridge the layer spacing thereby forming a monolayer SmA_1_ phase, and it is too long to allow two molecules to span the layer, as in the bilayer SmA_2_ phase. One possibility is that the structure is actually a bilayer where the molecules are tilted to form a smectic C_2_ phase.^[27]^ However, microscopy studies show the phase is uniaxial, and therefore the molecules are essentially upright within the layers and not tilted. Therefore, the lamellar phase does not possess mono‐ or bi‐layer structures, and possibly should have a structure with two layers of molecules that overlap with one another.

**Figure 11:**
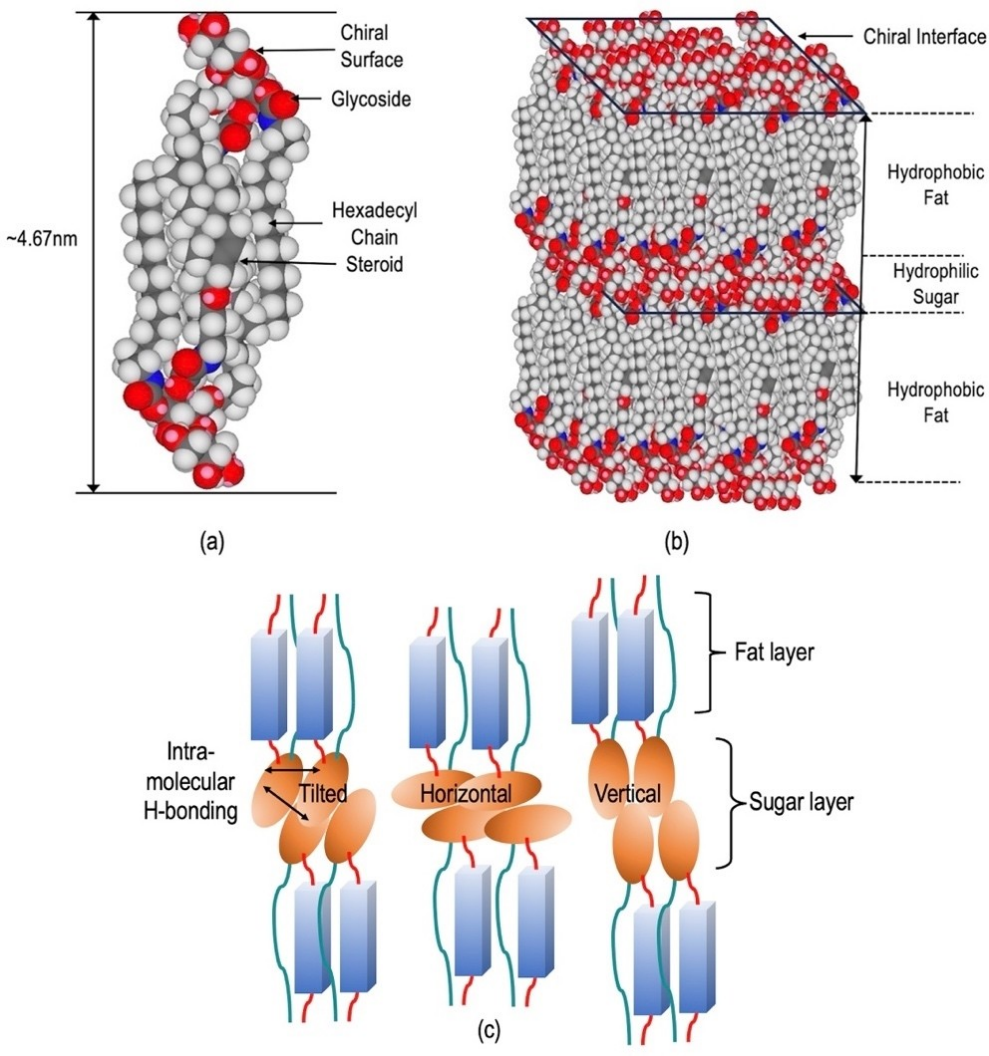
(a) A picture showing the antiparallel packing of two molecules of compound **6**, such that the hydrophobic regions overlap with one another in the middle of the packing. (b) A three‐dimensional sketch of the structure of the lamellar phase made up of the pairs of the molecules. The layer surfaces shown by the two parallelograms are chiral due to the packing of the disaccharide moieties together, via the formation of inter‐ and intra‐molecular H‐bonds. (c) Interfacial interactions between the disaccharide moieties in the lamellar assembly. Interactions; left, a tilted arrangement; middle, direct facial interactions; and right, interpenetration between layers.

If we consider a simple situation where two parallel molecules overlap with one another in order to span the layer, the overlap should be 4.67 nm. In order to determine how the molecules overlap we should first consider the parts of the molecules that interact with one another but not with other parts, thereby giving segregation to the packing. In the case of the materials the polar H‐bonding parts of the molecules will segregate from the non‐polar sections, i. e. sugars from fats.^[29]^ The aliphatic region was determined to be around 2.46 nm, and the extended polar region 1.02 nm. The closest approximation to do this is two disaccharide units and one aliphatic section, which add up to 4.5 nm, which is near to the value of the layer spacing of 4.67 nm measured from X‐ray scattering studies. This means that the lamellar structure has two molecular layers, where there is an overlap of aliphatic regions along the middle of the lamellar structure, with sugar moieties squeezed out or excluded[^30^, ^31^] to the surfaces of the lamellar assembly resulting in a mesophase that might be termed a smectic A_d_ phase. The basic assembly upon which the three‐dimensional mesophase structure is based is shown in Figure 11(a) for the packing of two molecules together.

A cartoon of the structure of the lamellar phase is shown in Figure 11(b). In this model the flexible aliphatic chain and the relatively rigid steroid unit would potentially overlap because of the flexibility and conformational variance of the aliphatic chain. Packing together of these units in the 3D structure would produce bands of non‐polar, hydrophobic fat, and bands of polar H‐bonding sugar moieties, as shown. On the other hand, the disaccharide units are not spherical in shape but more likely to be elliptical. They will also have strong intra‐ and inter‐molecular H‐bonding interactions, which will allow for various interpenetrated or face‐to‐face orientations and interactions (vertical, horizontal, and tilted) between the layers, as shown in Figure 11(c). The vertical interactions between the disaccharide units as shown indicate that the layer spacing for this molecular packing is the largest, and therefore closer to the measured value of lamellar spacing. However, not only will there be mixtures of H‐bonding interactions between the disaccharide units within the layers but also between the layers; and in addition, via interactions with the amide moieties. As shown in the picture, the layer spacing will be dependent on the mixed combinations of interactions, and as a result the H‐bonding layer will probably be diffuse, with the molecules in dynamic motion. Furthermore, there could also be the possibility of molecules of hydration which will increase the number and variety of the interfacial interactions between the layers, which will affect the width of the H‐bonding layer and its diffuse nature.

So far, we have not mentioned the stereochemical structures of the materials. The overall source of chirality lies in the disaccharide units and also in the steroid moieties. For a single molecular layer having a sugar‐fat‐sugar composition, the top and bottom surfaces will be decorated with H‐bonding chiral disaccharide units, thereby creating opposing chiral H‐bonding surfaces for the layer. Conversely, within the layers the side‐by‐side packing of the weakly interacting chiral steroidal hydrophobic units will have a tendency to form a lateral twist, as experienced in cholesteric phases. Furthermore, the addition of the layers to the top and bottom of the single layer will induce chiral interactions between the layer interfaces, thereby adding to the tendency to form a helix occurring parallel to, and within, the layer planes. Such structures have been found in thermotropic twist grain boundary smectic A phases,^[32]^ where lattices of screw dislocations have been found to facilitate the formation of a macroscopic helix composed of repeating blocks of the lamellar phase that rotate relative to one another along the helical axis, which could aid in the formation of bicontinous structures.^[8]^

**Figure 12:**
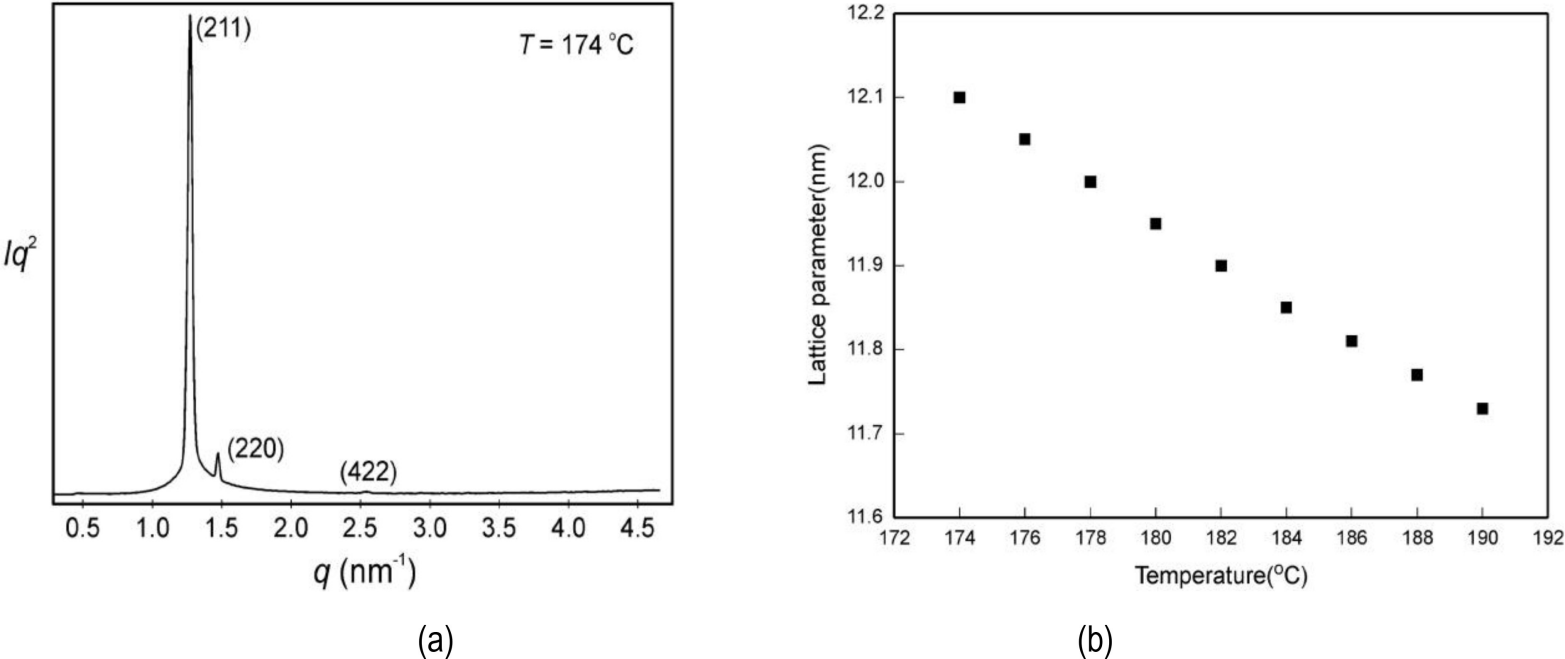
(a) Experimental SAXS reflections for compound **6** taken at 174 °C, and (b) the lattice parameters taken over a range of 174 to 190 °C for the cubic phase (intensities are shown in arbitrary units).

**Figure 13:**
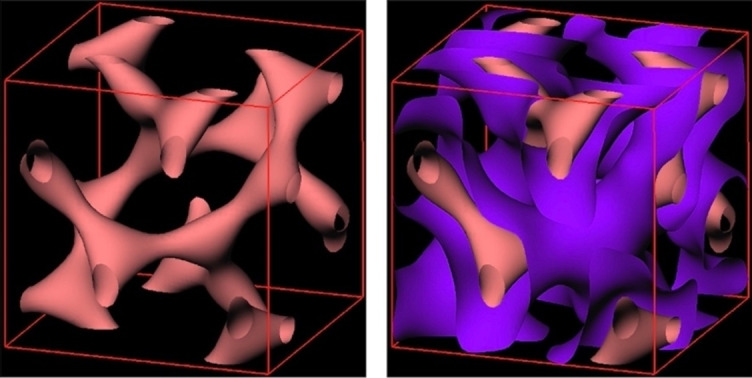
The gyroid lattice of the cubic phase of compound **6** showing two interwoven arrangements of the molecules produced via reconstruction of the electron density map obtained from the powder pattern at 174 °C. The red/pink isoelectron surfaces enclose the low electron density regions (fats), and the blue/purple ones the high electron density regions around the minimum surface (sugars).

The full temperature X‐ray scattering scan for **6** is shown in Figure 9(a), and confirms that the cubic phase exists over a temperature range of approximately 16 °C. Therefore, to elucidate the structure of the cubic phase, the diffraction results were analysed at the beginning of the phase at 174 °C, the trace for which is shown in Figure 12(a). The experimental and calculated *d*‐spacings of the three observed SAXS reflections were fitted to (211), (220) and (422) peaks, thereby giving a cubic structure with $Ia\bar{3}d$ symmetry. The experimental and calculated *d*‐spacings of the diffraction peaks were compared at temperatures of 174 and 190 °C respectively in Table 2, along with the intensity values (Lorentz and multiplicity corrected). The dominant (211) and (220) diffraction peaks for the cubic phase are related to the structure of a double gyroid, bicontinuous, cubic phase, with no indication from SAXS for the presence of any other phase being present, even though there was a slight compression of the lattice parameter from *a*_cub_=12.10 nm at 174 °C to 11.73 nm at 190 °C. Moreover, the change in the lattice parameter *a*_cub_ (nm) of the cubic phase from 174 to 200 °C was shown to fall almost linearly with respect to temperature, as shown in Figure 12(b). Using the predicted symmetry for the cubic phase, a similar correlation of the results between the experimental and calculated data was found at the temperature of 190 °C, as shown in Table 2. Furthermore, this confirmed there were no additional cubic phases present from 174 to 190 °C, and that the cubic phase with $Ia\bar{3}d$ symmetry was the only mesomorphic modification formed. The structure of the cubic phase, therefore, has a gyroid lattice with two interwoven defect arrangements of the molecules as shown in Figure 13, produced by reconstruction of the electron density map obtained from the X‐ray scattering powder pattern at 174 °C.

**Table 2:** Experimental and calculated *d*‐spacings of the observed SAXS reflections for the cubic phase of compound **6** at 174 °C (top) and 190 °C (bottom). All intensity values are Lorentz and multiplicity corrected.

		Temperature 174 °C		
(*hkl*)	*d*_obs._ – spacing (nm)	*d*_cal._ – spacing (nm)	intensity	phase
(211)	4.94	4.94	100.0	0
(220)	4.27	4.28	6.9	0
(422)	2.47	2.47	0.3	π
*a*_cub_=12.10 nm				

		Temperature 190 °C		
(*hkl*)	*d*_obs._ – spacing (nm)	*d*_cal._ – spacing (nm)	intensity	phase
(211)	4.79	4.79	100.0	0
(220)	4.14	4.15	52.1	0
(422)	2.40	2.39	0.1	π
*a*_cub_=11.73 nm				

Compound **6** was found to exhibit just lamellar and cubic mesophases, but another lower temperature mesophase (X) was found in other compounds. Therefore, we now examine compound **5**, which by POM exhibits the following phase sequence of K 120 X 153.6 cubic 180 lamellar 220 °C Iso Liq. From small angle X‐ray scattering experiments, the sequence for **5** was found to be:

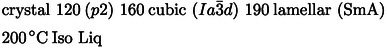




**Figure 14:**
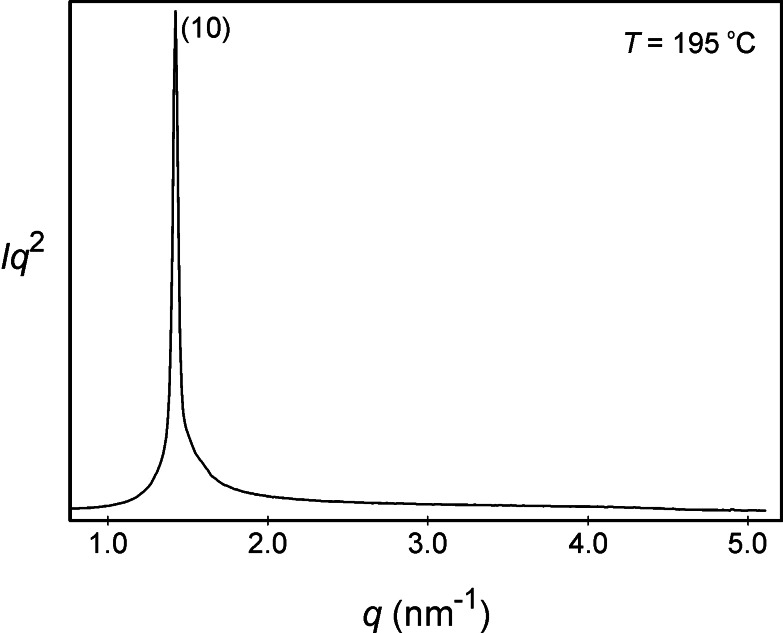
X‐ray scattering profile for the lamellar phase of compound **5**.

The experimental and calculated *d*‐spacings for the observed SAXS reflections, and the lattice parameters for each mesophase of compound **5** are given in the following sections starting with the lamellar phase where the intensity values are Lorentz and multiplicity corrected, as was the case for compound **6**. Thus, Figure 14 shows the X‐ray scattering profile and the powder diffraction pattern for the lamellar phase of **5**, recorded at a temperature of at 195 °C (where the intensities are in arbitrary units). Table S2 in the SI gives the experimental and calculated *d*‐spacings for the observed SAXS reflections of the lamellar phase for **5** also at 195 °C. The layer spacing was determined to be 4.42 nm, at 195 °C, which compares with a value of 4.67 nm at 200 °C for compound **6**.

Given that the disaccharide and cholesteryl moieties are the same for both **5** and **6**, it appears that the slightly lower value for the *d* spacing of **5** is due to the only variable, i. e. the length of the lateral aliphatic chain. In the case of **5** the difference from **6** is 0.25 nm, or 0.125 nm per molecule in the packing (pairing), which is appropriate.

**Figure 15:**
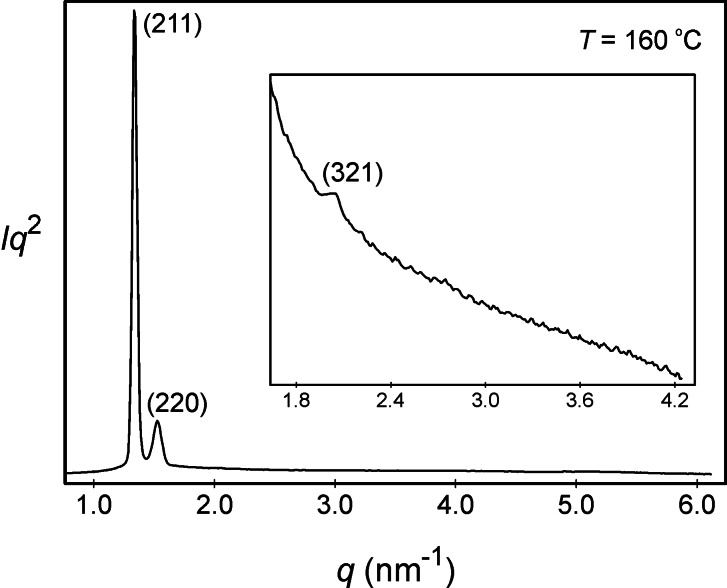
Powder diffraction pattern of the cubic $Ia\bar{3}d$ phase of compound **5** recorded at 160 °C.

For the cubic phase of compound **5**, the powder diffraction pattern was generated from reflections from the (211), (220), and (321) planes as shown in Figure 15, the main peak arising from the (221) plane, but with the other two reflections being relatively weak. In comparison compound **6** also has reflections from the (211) and (220) planes, but the third plane differs from the (321) plane. Table S6 in the SI lists all of the *d*‐spacings of the observed for the SAXS reflections for **5**, with the lattice dimension for the cubic phase *a*_cub_ having a value of 11.48 nm, which is smaller than the results obtained for **6**.

Thus, the reconstruction of the electron density map obtained for **5** from the powder pattern at 160 °C showed that the lattice of the cubic phase was gyroid with two interwoven arrangements of the molecules in a similar way to the organization shown in Figure 13. Thus, the structure of the cubic phase of **5** is consistent with that of **6** and also for the homologous family of materials.

**Figure 16:**
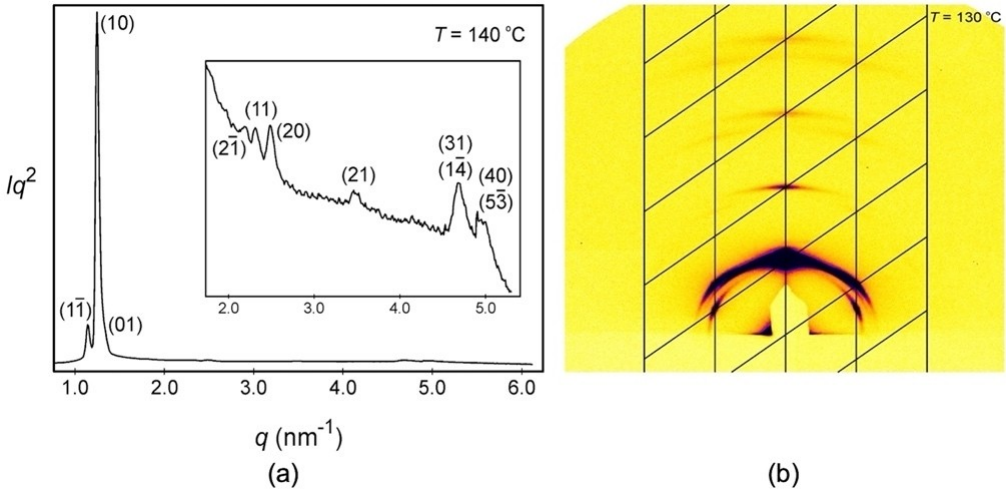
(a) X‐ray scattering profile at a temperature of 140 °C for the oblique phase X.(b) 2‐D powder diffraction pattern for mesophase X of compound **5** at 130 °C.

We now turn to the lowest temperature mesophase of compound **5**, the X phase; consisting of modulated layers, with a 2‐D lattice and *p*2 plane group symmetry. Firstly, the X‐ray scattering profile, which is shown in Figure 16(a), was generated at a temperature of 140 °C for the oblique phase of **5**, and the 2‐D powder diffraction pattern for the oblique phase X, shown in Figure 16(b) at 130 °C. In comparison to the cubic phase the oblique phase exhibits more Miller planes, indicating that the phase has a more extensive and organized structure, or a lesser degree of thermal fluctuation. Experimental and calculated *d*‐spacings for the observed SAXS reflections of the oblique phase of **5** at 140 °C are given in Table 3 and the SI.

**Table 3:** Experimental and calculated *d*‐spacings of the observed SAXS reflections of the oblique phase in compound **5** at 140 °C. All intensity values are Lorentz and multiplicity corrected.

(hk)	d_obs._ – spacing (nm)	d_cal._ – spacing (nm)	intensity	phase
(1‐1)	5.50	5.50	13.0	π
(10)	5.04	5.04	100.0	π
(01)	4.75	4.75	14.9	π
(2‐1)	2.90	2.95	0.1	/
(11)	2.71	2.73	0.2	/
(20)	2.52	2.52	0.5	/
(21)	1.80	1.82	0.2	/
(31)	1.34	1.35	0.8	/
(1‐4)			0.8	/
(40)	1.26	1.26	0.8	/
(5‐3)			0.8	/

a=6.33 nm, b=5.97 nm, *γ*=127.3°.

**Figure 17:**
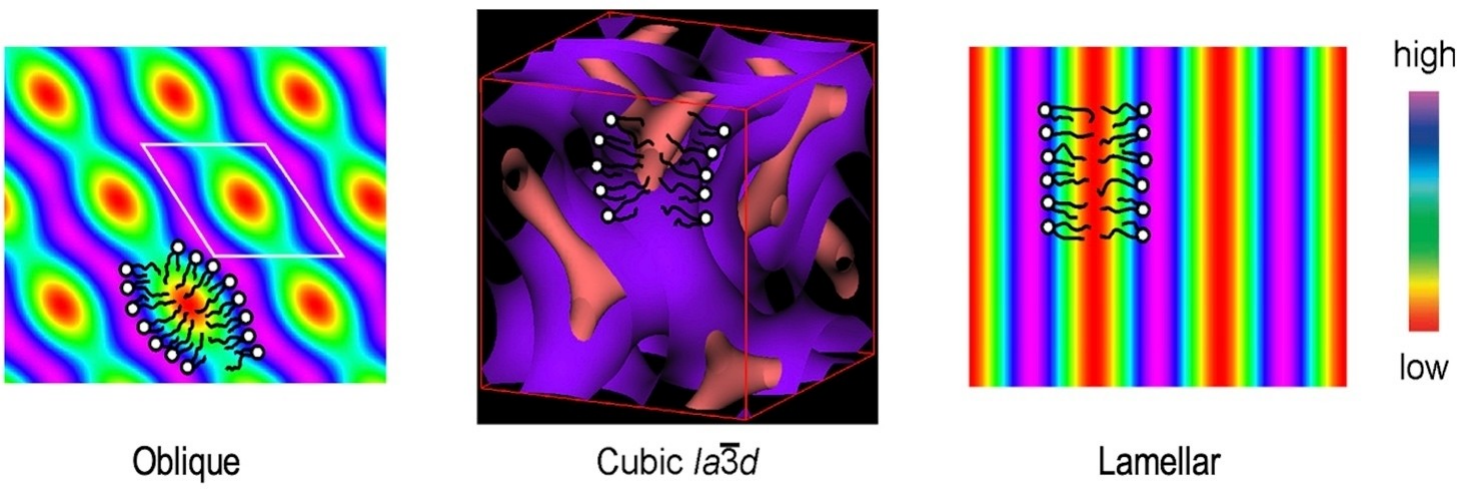
Reconstructed electron density maps for the oblique p/2, cubic, and lamellar phases of compound **5** derived from their associated powder patterns. The hydrophilic head groups, and hydrophobic tails are located in areas of high electron density (blue/purple) and low electron density (red/pink).

Taking the powder X‐ray scattering pattern in Figure 16(a) and reconstructing the electron density map for the oblique *p*2 phase at 140 °C, the structural pattern for the locations of the molecules was obtained, as shown in Figure 17. The blue modulations/waves give the locations of the parts of the molecules that are electron rich, which in this case are hydrophilic head groups. The red areas indicate the locations of the electron poor hydrophobic regions, which for this case are the aliphatic lipid parts. For compound **5** the three mesophases, oblique, cubic, and lamellar, were observed and their structures analysed, thereby giving the possibility of reconstructing electron density maps for all three for comparison; they are pictured together in Figure 17, with sketches of the molecules overlaying the coloured high and low electron density areas.

In addition to compounds **5** and **6**, compounds **2** to **4** and **7** were also investigated by SAXS. Their phase sequences and approximate transitions temperatures (°C) upon heating are collated in Table 4. Details of the structures of the oblique, cubic, and lamellar phases fell into the same forms as for compounds **6** and **7**. Full details of the melting behaviours, mesophase types, and lattice parameters for compounds **2** to **7** are given in the Supplementary Information (SI), Tables S3 to S8 inclusive.

**Table 4:** The transition temperatures (°C) determined on heating by X‐ray scattering for compounds **2** to **7**.

Cpd	n	Transition Temperatures (°C) on Heating
**2**	8	Cr 100 Lamellar 220 Iso Liq
**3**	10	Cr 140 Oblique 150 Cubic 180 Lamellar 200 Iso Liq
**4**	12	Cr 110 Oblique 140 Cubic 180 Lamellar 200 Iso Liq
**5**	14	Cr 120 Oblique 160 Cubic 190 Lamellar 200 Iso Liq
**6**	16	Cr 172 Cubic 190 Lamellar 210 Iso Liq
**7**	18	Cr 140 Oblique 160 Cubic 190 Lamellar 210 Iso Liq

### 3.4 Simulations and Modelling of the Layered Structure of Compound 6

For the analysis of the lamellar structure of compound **6**, the molecular length was determined via DFT and also replicated in ChemDraw using MOPAC energy minimization and found to be approximately 34.2 Å. Packing of the molecules side by side in the lamellar phase means that it has an interdigitated structure where the overlap of the molecules is about 10 Å, which corresponds roughly in size to the disaccharide units.

Given that the experimental results indicate upon cooling the liquid phase of compound **6** is smectic and there may be pre‐transitional organization, it seemed appropriate to begin simulations on mesophase formation starting with preliminary layering in the liquid state. Computational details are given in the SI, and the results are summarised below.

**Figure 18:**
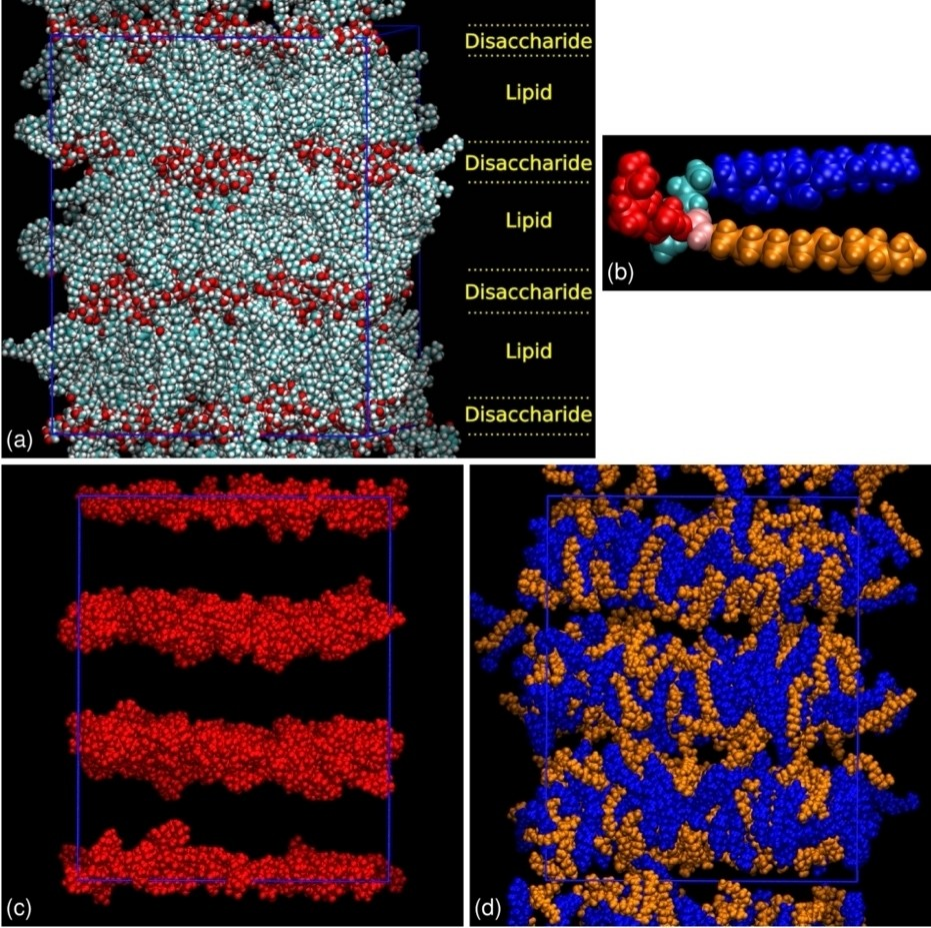
Snapshots from the final frame of a 100 ns MD simulation of compound **6** at 473 K: (a) all atoms included with standard atom colours, and with the alternating layers of disaccharides and lipids labelled; (b) a single molecule of compound **6** extracted from the simulation coloured by moiety, with the disaccharide unit in red, the steroidal unit in blue, the flexible lateral aliphatic chain in gold, the disaccharide‐steroidal linking group in cyan, and the disaccharide‐aliphatic chain linking group in pink; (c) only disaccharide units displayed in red; (d) steroidal units and flexible lateral aliphatic chains displayed in blue and gold, respectively. The simulation box is shown in blue.

A preliminary molecular dynamics (MD) simulation of the layering of compound **6** was carried out for 100 ns on a pre‐formed bilayer structure at 473 K, as described in detail in the SI (Simulation Details, Figure S3). The pre‐formed bilayer structure was found to be stable, remaining essentially unchanged throughout the MD simulation run. Figure 18(a) shows a snapshot from the final frame of the simulation, using standard colour coding for all the atom types. Figure 18(b) shows a molecule of **6** with five whole moieties coded by single colours, and Figures 18(c) and (d) show different displays of the full final frame using selections of these colour‐coded moieties.

The simulated system comprised layers of sugar units, with the steroid and alkyl chains interdigitated between them in a disordered fashion, as shown in Figures 18(a), (c) and (d). The simulation box contained three layers perpendicular to the *z*‐axis and the layers extended across the *xy*‐plane, as seen most readily by the view of the sugar units shown in Figure 18(c). The z‐dimension of the simulation box elongated slightly during the simulation run and was 11.16 nm at 100 ns, giving an approximate layer spacing of ca. 3.72 nm, and with the sugar and lipid layers having widths of ca. 1.3 and 2.5 nm, respectively. This overall layer spacing is smaller than the experimental value of 4.67 nm, but it is a reasonable first simulation attempt from an artificial layered structure that will retain some paramorphotic order. The overall layer spacing had expanded slightly (by ca. 0.3 nm) during the simulation run, suggesting that extended simulation times might give results nearer to those obtained from SAXS. Additional MD simulations using different compression protocols to create the starting bilayer structure indicated that the layer spacing was essentially determined at the compression stage (details in SI); any changes during the full simulation runs were small, which is consistent with the layers being stable once formed.

Figures 18(c) and (d) give a better view of the distributions of the molecular moieties within the layer organization, and hence the degree of nanosegregation. The red segments show layers of H‐bonding disaccharide units, which are segregated from one another by lipids composed of semi‐rigid blue steroidal units and flexible lateral aliphatic gold‐coloured chains. This dispersion of flexible and semi‐rigid moieties may be expected to disturb the flow across the aliphatic layers, which in turn may possibly affect the anisotropic viscoeleastic properties of the system. Furthermore, as a consequence, the segregation is likely to depend upon on the various polar/non‐polar, H‐bonding/non‐H‐bonding, and rigid/semi‐flexible interactions in the stratified structure. This is shown more clearly in Figure 18(c) where all but the carbohydrate units are removed, revealing a layer that is dependent on polar‐non‐polar and hydrogen bonding interactions. Perpendicular to the layers these strata repeat as carbohydrate bandwidths of relatively well‐defined thickness, but where there is no molecular periodicity, as shown by Figure 18(a).

Therefore, there is long range ordering of a stratified structure, which is inconsistent with the sinusoidal modulated density waves found for smectic mesophases, and in particular the smectic A phase as described by Leadbetter.^[33]^ Consequently, more realistic simulations using much larger box sizes possessing greater numbers of molecules, and simulations on cooling from the isotropic liquid such that the paramorphotic behaviour of a compound may be required.

## 4 Discussion

In his 1962 textbook^[34]^ Gray devotes a chapter on transition temperatures for homologous series which was focused on nematic and smectic mesophases. In Figure IX.2 in this book numerous homologous series are depicted, with each showing smooth curves for transition temperatures of mesophases as a function of chain length. Subsequently, numerous studies on nematic and smectic phases have also shown similar behaviours, with some exhibiting odd‐even trends in temperature as a function of aliphatic chain length.[^35^, ^36^, ^37^, ^38^] Fewer investigations on homologous series showing odd‐even effects have been made on materials exhibiting nematic and columnar phases,^[39]^ and even less on materials possessing nematic, smectic, columnar and cubic phases.^[40]^ And as noted in the introduction there are very few individual examples of materials that exhibit different properties on heating versus cooling.

**Figure 19:**
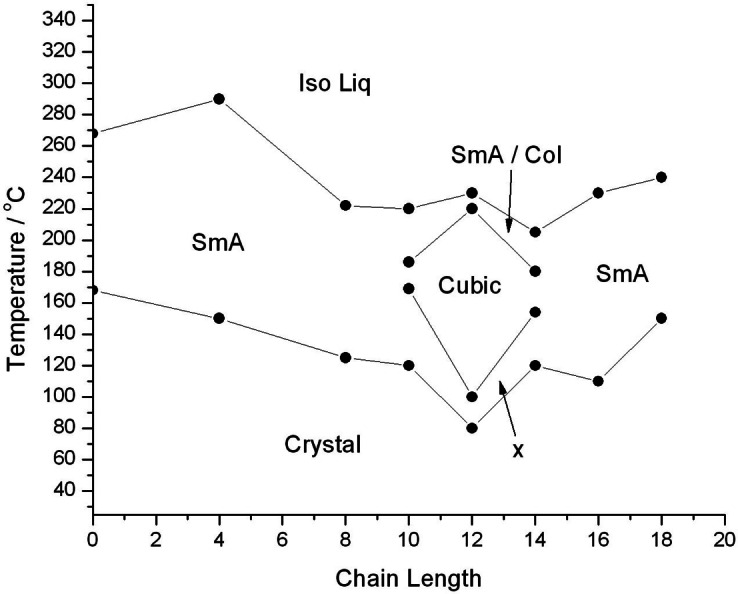
The transition temperatures (°C) of various phases observed upon heating for the homologous series of of glycosteroids as a function of lateral aliphatic chain length (n).

In Figure 19 we show the transition temperatures as a function of lateral aliphatic chain length for the glycosteroids determined by POM on heating. As we ascend the series the lateral chain extends, becoming longer than the hydrophobic steroidal unit. Thus, as the series progresses the hydrophobic‐hydrophilic balance changes. Interestingly, at the beginning of the homologous series the homologues exhibit lamellar mesophases, and again at the end of the series studied. In between there is a change over with the introduction of the sequences of columnar, cubic and oblique phases on lowering the temperature, appearing as a “puddle” in the middle of the series. The higher temperature mesophase is probably lamellar as we see upon cooling, the second is the more ordered columnar phase, then this is followed by the 3D ordered cubic phase and the most ordered oblique phase, which would be expected for a heating cycle for a normal liquid crystal melting from the solid state to the liquid through a number of mesophases that are becoming less ordered upon heating. On the cooling cycle we find that the lamellar phase is formed and retained down to around room temperature when glassification occurs, and the other phases are not observed at all no matter what the cooling rate was. This potentially indicates that the other phases are subject to supercooling, which matches with our observations. Thus, between the solid state and the amorphous liquid there is a specific change in molecular ordering and packing to generate intermediary mesophases that may be 3D for cubic or oblique mesophases, or 2D for columnar phases, and 1D for lamellar, or variations thereof. This is no different in principle from mesophases formed by like rod‐like molecules as described for smectic and nematic phases,^[33]^ except for the molecules having different shapes, most notably broader or wedge‐like as seen for the molecules in Figure 1 and in references,[^38^, ^40^] that might induce curvature into the molecular packing.

An interesting question is why does the “bubble” occur for the homologues with n=10–14? Firstly, the values for n are all even, and therefore there are no odd‐even effects, associated with the jumps in the changes in transition temperatures which can be as much as 30 degrees. Hence, given the purities of the products, these changes are real effects and not artifacts, such as due to impurities. Secondly the introduction of cubic phases etc., occur when lateral aliphatic chains start to become greater in length than the steroidal unit, thereby broadening the molecular architecture and distorting the molecular shape. Thirdly, as n is increased, the lateral chain becomes longer and the molecular architecture reverts back to being more rod‐like. Fourthly, for low values of n the steroidal units dominate the hydrophobic unit, and the shape does not initially change and remains rod‐like because the steroidal unit is not particularly flexible. Thus, only between n =10 to 14 is there change in shape competition, which will also affects the hydrophobic/hydrophilic balance that affect the formation of mesophases.

## 5 Conclusions

This article describes the syntheses of seven homologues of the following family of *N*‐{4‐[(3β)‐cholest‐5‐en‐3‐yloxy]butyl}‐2‐({2‐*O*‐[(alkylamino)carbonyl]‐4‐*O‐*β‐D‐glucopyranosyl‐α‐D‐glucopyranosyl}oxy) acetamides, where the alkyl chain length (C_n_H_2n+1_) was varied in even numbers of methylene units (n=4 to 18).

The homologues exhibited liquid‐crystalline mesophases, determined initially by POM, ranging from lamellar to columnar, cubic and oblique, with calorimetry achieved by DSC showing that the enthalpies of the phase transitions were extremely small. Standard heating and cooling cycles for liquid crystals composed of rod‐like molecules normally show reversibility occurring over 1 to 2 °C, however, for the homologous series reversibility was not usually observed and instead heating often exhibited the following succession:

crystal→oblique→cubic→columnar→lamellar→liquid

but on cooling the reverse sequence:

liquid→lamellar→glass

was obtained possibly due to supercooling of the lamellar phase preventing the formation of the other more ordered mesophases. These sequences potentially indicate that on heating there is melting of 3D ordered oblique and/or cubic mesophases to 2D ordering of columnar, then to 1D ordering of lamellar phases, with everything to be gained by increasing disordering of the molecules as the temperature increases. Conversely, on cooling the lamellar phase is retained, possibly due to the potential for the low enthalpies of transition providing no benefit to molecular reorganization.

Across the homologous series there is another change in properties. For low and high values of n only simple phase sequences were observed, often with only the lamellar phase appearing, whereas near to the centre of homologous series more complex sequences were observed, which indicates specific architectural changes were occurring with increasing values of n. For low n the lateral aliphatic chain did not protrude beyond the steroidal unit, whereas for high values of n the reverse was the case. The cross‐over of the properties appeared to be at a point where the molecular shape changed, and hence the packing arrangements also differed, possibly from packing of rod‐like molecules for low and high n via the packing of wedge‐like molecules inducing curvature, which supported the formations of the columnar and cubic phases.^[40]^ This was possibly accompanied by changes to the hydrophobic/hydrophilic balance as the proportion of non‐polar units in the molecular architecture is increased as n increases.

Initial investigations of the type we reported on would not have been normally observed in standard studies because for characterizations of mesomorphic behaviour one would expect reversibility of phase transitions. The large differences in transition temperatures on heating and cooling, and the possibility of supercooling, would usually point to impurities. However, the high level of purities of the products and the resyntheses of some of the materials producing matching products with similar to almost exact properties, rules out such artefacts, indicating that the dependency of molecular packing on shape and the extent of the weak crystallinic ordering of some of the phases suppresses typical reversibility.^[41]^

## Experimental

*Synthesis of Target Materials*: The structures of all intermediate and final compounds were fully characterized by NMR spectroscopy and mass spectrometry. The written details of the analyses along with the yields for the final products are given in the SI with the spectra for the final materials. The position of the carbamoyl chain at O‐2 in the final compounds was found to be unambiguous as it arises from the reaction of the 2‐monohydroxy precursor **11** with all other positions protected, itself arising from the opening of the lactone. This was also confirmed by 2D NMR HMBC spectroscopy through observation of the correlations between the H2 of the sugar and the carbamoyl group as shown by Figure S10 in the SI.

*Polarized Light Microscopy*: As with most studies of liquid crystal phase transitions,[^23^, ^42^, ^43^] the initial phase behaviour was evaluated using thermal polarized light microscopy (POM) utilizing a Zeiss Axioskop 40Pol microscope using a Mettler FP82HT hot‐stage controlled by a Mettler FP90 central processor.

*Calorimetry*: Differential scanning calorimetry was performed on a Mettler DSC822e fitted with an autosampler operating with Mettler Stare software and calibrated before use against an indium standard (onset=156.55±0.2 °C, ΔH=28.45±0.40 J g^−1^) under an atmosphere of dry nitrogen. DSC and POM were used in conjunction with one another to determine phase transition temperatures and identify mesophase types.

DSC was exploited in the determination of thermodynamic melting points of pristine products, whereas kinetic recrystallization temperatures were expected to be variable and subject to supercooling. For each material, it was produced in its crystalline form via recrystallization as part of the synthetic pathway. The first DSC heating cycles of 5, 10, or 20 °C min^−1^ for individual specimens subsequently gave the melting point of a material, whereas the cooling cycles gave various recrystallization and/or supercooling temperatures. These were used to avoid possible issues of paramorphosis associated with defect textures^[44]^ when observed via microscopy studies.[^23^, ^42^, ^43^] Consequently, the combination of POM and DSC results obtained upon cooling whereby paramorphosis could be observed, then allowed for mesophase classification. Subsequent heating and cooling cycles were made by taking each material to the clearing point and cooling it back into the highest temperature mesophase, thereby also allowing for observation of any decomposition.

It should be noted that initial thermograms across the family of materials sometimes appeared to be of poor quality, however, they correlated directly with respect to materials that were resynthesized, which indicated that reprepared materials have very similar purities/impurities. In addition, the resulting thermograms were affected by the order of each phase transition (first order, second order or a combination of the two), reproducibility from different samples, relative scan rates, heat and cool cycles, and observed thermal decomposition. The variation in the baselines to each scan can indicate impurity levels and decomposition as observed for some of the materials, which indicated susceptibility for stability in further studies, such as by SAXS.

*X‐ray Diffraction*: Powder X‐ray investigations were carried out with a Guinier film camera, samples in glass capillaries (Ø of 1 mm) in a temperature‐controlled heating stage, quartz‐monochromatized Cu Kα radiation, 30–60 min exposure time and calibration with the powder pattern of Pb(NO_3_)_2_. Small angle X‐ray diffraction was performed using a Bruker D8 Discover equipped with a temperature controlled, bored graphite rod furnace. The radiation used was copper Kα (λ=0.154056 nm) from a 1 μS microfocus source. Diffraction patterns were recorded on a 2048×2048 pixel Bruker VANTEC 500 area detector set at a distance of 121 mm from the sample. Samples were filled into 1 mm capillary tubes and aligned with a pair of 1 T magnets, with the field strength at the sample position being approximately 0.6 T Diffraction patterns were collected as a function of temperature and the data processed using Matlab.

*Computational Modelling*: Fully atomistic MD simulations were performed using GROMACS, and the GAFF force field. The MD simulations were run from a pre‐formed bilayer structure comprising six layers of 8 x 8 molecules of compound **6** (384 molecules in total), at a temperature of 473 K, and using anisotropic pressure coupling at 1 atm. Full details are given in the SI.

## 6 Disclosure Statement

No potential conflict of interest was reported by the authors.

## 7 Funding

Financial support from to CNRS and MESRI for is gratefully acknowledged. The authors also thank the General Directorate for Scientific Research and Technological Development (DG‐RSDT), Algerian Ministry of Scientific Research, for a grant to FAR, the Fundação para a Ciência e a Tecnologia (FCT) for grant to NMX (CEECIND/03881/2018), and the China Scholarship Council program for a scholarship to XY. The authors are also indebted to financial support from the EPSRC (Platform Grant EP/D055261/1, Core Capability Grant EP/K039660/1, and Standard Grant EP/M020584/1).

## Conflict of Interests

The authors declare no conflict of interest.

8

## Supporting information

As a service to our authors and readers, this journal provides supporting information supplied by the authors. Such materials are peer reviewed and may be re‐organized for online delivery, but are not copy‐edited or typeset. Technical support issues arising from supporting information (other than missing files) should be addressed to the authors.

Supporting Information

## Data Availability

The data that support the findings of this study are available in the supplementary material of this article.
