# Supplementary material for: The Unusual Mesophases and Properties Exhibited by a Family of Glycosteroids
Source: Chemistry. 2025 Jan 31;31(11):e202403678. doi: 10.1002/chem.202403678 (PMC11840659; doi:10.1002/chem.202403678)
Supplement: Supplementary file 1 — Supporting Information [file CHEM-31-e202403678-s001.pdf]

Chemistry–A European Journal

Supporting Information

The Unusual Mesophases and Properties Exhibited by a Family of Glycosteroids

Fahima Ali-Rachedi, Nuno M. Xavier, Xiaoyang Yue, Stéphane Chambert, Feng Liu, Laurence C. Abbott, John N. Moore, Xiangbing Zeng,* Stephen J. Cowling,* Yves Queneau,* and John W. Goodby*

Supplementary information
The Unusual Mesophases and Properties Exhibited by a Family of Glycosteroidal Bolaphiles

Characterization of Mesophase Structures

1.1 Microscopy and Calorimetry: Polarized optical microscopy was performed on a Zeiss Axioskop 40Pol microscope using a Mettler FP82HT hot-stage controlled by a Mettler FP90 central processor. Photomicrographs were captured via an InfinityX-21 MP digital camera mounted atop of the microscope. Differential scanning calorimetry was performed on a Mettler DSC822e fitted with an autosampler operating with Mettler Stare software and calibrated before use against an indium standard (onset = 156.55 ± 0.2 °C, ΔH = 28.45 ± 0.40 Jg-1) under an atmosphere of dry nitrogen.

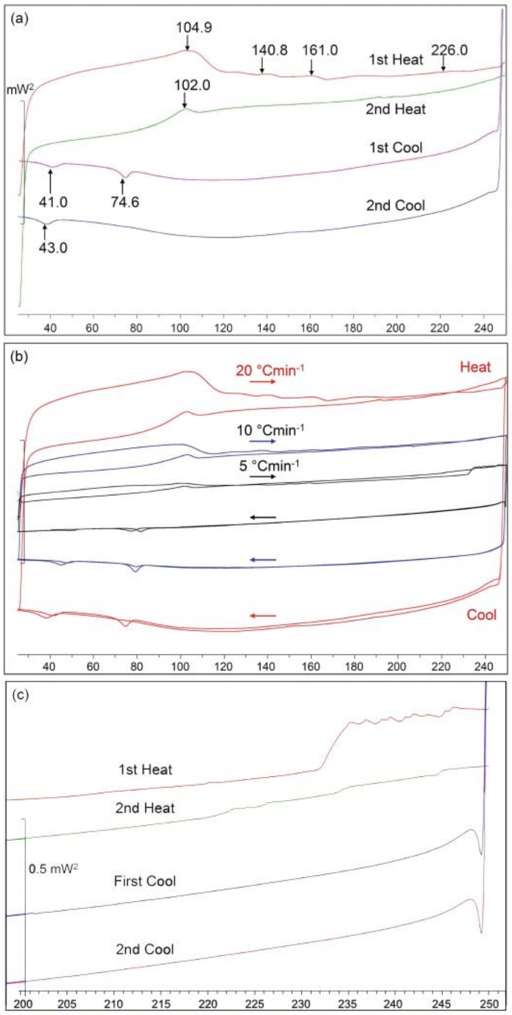

Figure S1: Differential scanning calorimetric thermograms ($mW^2/°C$) for the hexadecyl substituted (**6**, n = 16) glycosteroidal bolaphile**.** (a) Shows first and second heating and cooling traces taken at rates of $20°Cmin^{-1}$; (b) Shows scans taken at 20, 10 and 5°C min^{-1} on the first and second heating and cooling cycles; and (c) Shows an expansion of figure 7(b) near to the clearing point for compound **6**.

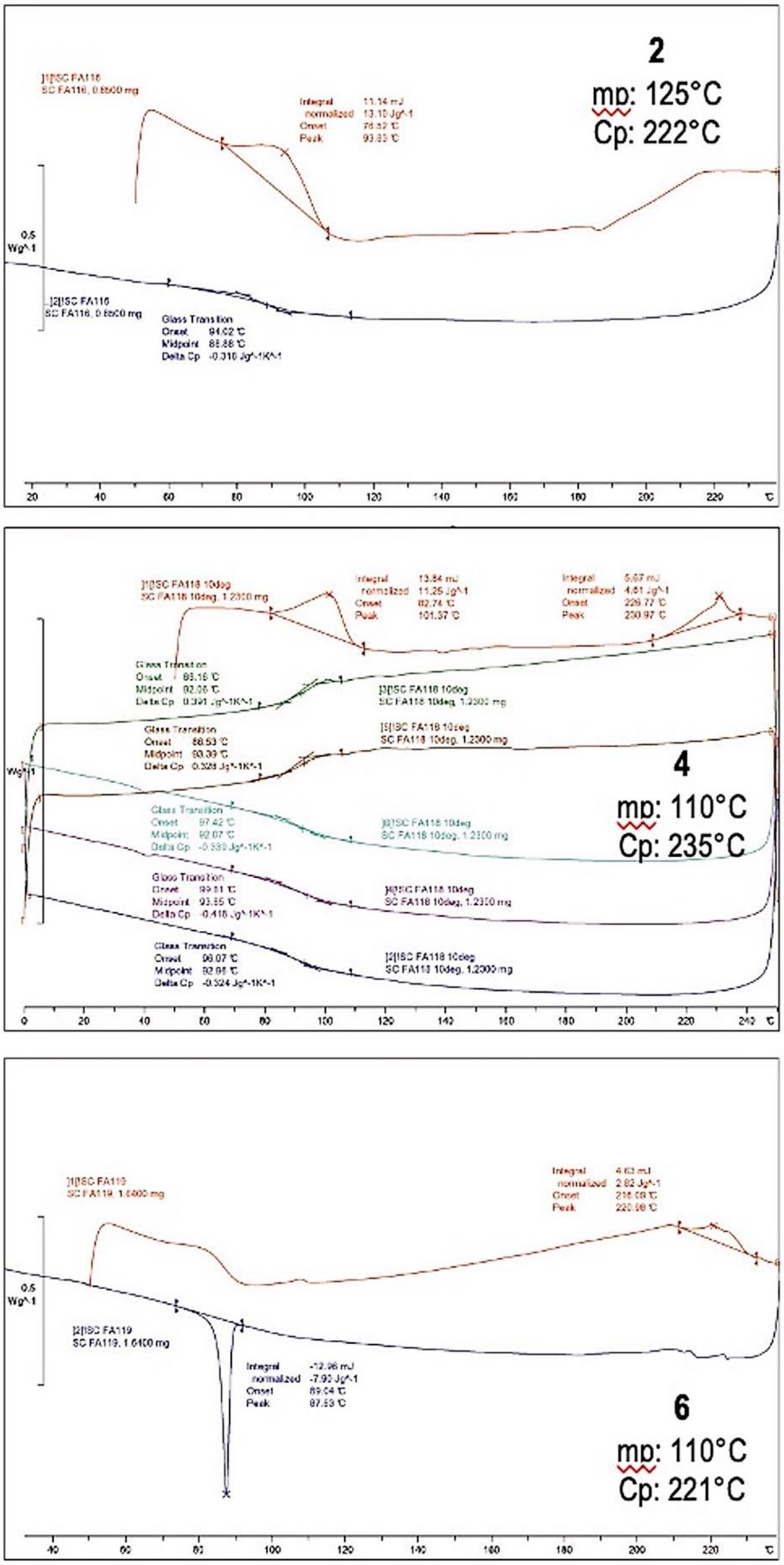

Figure S2: Comparison of differential scanning calorimetric thermograms (mW^2/°C) for three members of the family; **2** (n=8), **4** (n=12), and **6**(n= 6) from top to bottom. The heating and cooling traces taken at rates of 20°Cmin^{-1}.

Computational Modelling: Quantum chemical calculations were performed using the Gaussian 09 revision e.01 suite of programmes.

Simulation Details

The structure of compound **6** was optimised at the AM1 level [1] and then at the B3LYP/6-31++G(d,p) level[2,3], both using Gaussian 16 [4]. Electrostatic potentials were then calculated at the HF/6-31++G(d,p) level using Gaussian 16, before using AmberTools 19 [5] to generate a set of RESP [6] atomic charges and a set of GAFF version 1.81 forcefield parameters [7]. The forcefield was then converted using acpype.py [8] and molecular dynamics (MD) simulations were performed using GROMACS 2019.3 [9–15].

The initial simulation of the layering was achieved by using a pre-formed bilayer structure, generated as a spaced-out, ordered grid of 6 layers of 8 × 8 molecules (384 molecules in total), with molecules in alternate layers pointing “up” and “down” relative to the *z* axis; each molecule was randomly rotated about the *z* axis. This approach gave a basic bilayer structure with the layer normal along the *z*-axis but with space around each molecule, as shown in Figure S3.

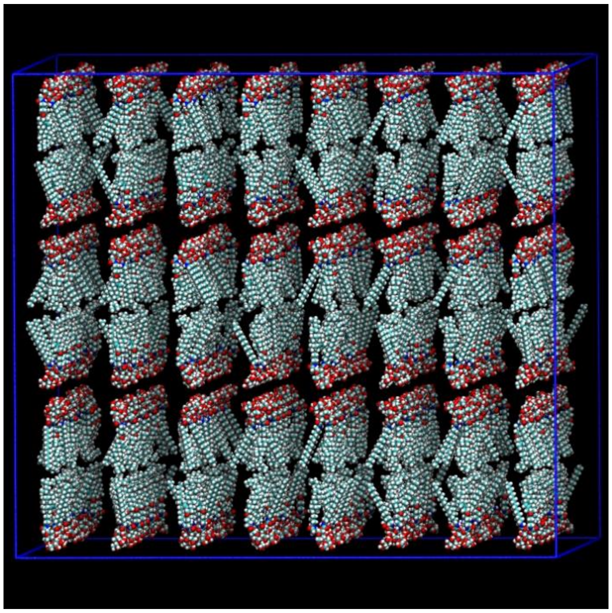

Figure S3: Pre-formed bilayer structure of compound **6**, with standard atom colours.

Simulations were run using 2 fs steps with all bond lengths constrained using the LINCS algorithm [16,17], and periodic boundary conditions were used in all directions. Long-range electrostatic interactions were handled using the particle mesh Ewald method [18] with a cut-off of 1.2 nm, and the same cut-off was used for van der Waals interactions; dispersion corrections for energy and pressure were used.

The pre-formed bilayer structure was compressed rapidly for 7 ps using a Berendsen barostat [19] with a nominal pressure of 50,000 bar and a 4-ps time constant to achieve a condensed-phase density; the temperature was held at 20 K using a velocity-rescaling thermostat [20] with a 1-ps time constant to minimise molecular diffusion during the compression. The system was then equilibrated at 473 K using a velocity-rescaling thermostat with a 1-ps time constant and at 1 bar using a Berendsen barostat with a 4-ps time constant.

The full MD simulation was then run at 473 K using a Nosé-Hoover thermostat [21,22] with a 1-ps time constant and anisotropic pressure coupling at 1 atm using a Parrinello-Rahman barostat [23,24] with a 4-ps time constant. The

simulation was run for 100 ns, and the simulated system remained essentially unchanged over this time, with a density of ca. 1020 kg m^{-3}. The simulation trajectories were visualised using VMD 1.9.4 [25].

X-ray Diffraction: Powder X-ray investigations were carried out with a Guinier film camera, samples in glass capillaries (Ø of 1 mm) in a temperature-controlled heating stage, quartz-monochromatized Cu Kα radiation, 30–60 min exposure time and calibration with the powder pattern of $Pb(NO_3)_2$. Small angle X-ray diffraction was performed using a Bruker D8 Discover equipped with a temperature controlled, bored graphite rod furnace. The radiation used was copper Kα (λ = 0.154056 nm) from a 1 μS microfocus source. Diffraction patterns were recorded on a 2048x2048 pixel Bruker VANTEC 500 area detector set at a distance of 121 mm from the sample. Samples were filled into 1mm capillary tubes and aligned with a pair of 1T magnets, with the field strength at the sample position being approximately 0.6T Diffraction patterns were collected as a function of temperature and the data processed using Matlab.

Tables **S1** and **S2** give the layer spacings for compound **6** and **5** respectively, whereas Tables **S4** to **S8** give the phase types and lattice parameters determined by SAXS for compounds **2-7**.

Table S1: Experimental and calculated *d*-spacing of the observed SAXS reflection of the lamellar phase for compound **6** at 200°C. (The intensity value is Lorentz and multiplicity corrected).

(*hk*)	$d_{obs.}$ – spacing (nm)	$d_{cal.}$ – spacing (nm)	intensity	Phase
(10)	4.67	4.67	100.0	π
d = 4.67 nm				

Table S2: Experimental and calculated d-spacings for the observed SAXS reflections of the lamellar phase of compound **5** at 195°C. (Intensity values are Lorentz and multiplicity corrected.)

(*hk*)	$d_{obs.}$ – spacing (nm)	$d_{cal.}$ – spacing (nm)	intensity	phase
(10)	4.42	4.42	100	π
d = 4.42 nm				

Table S3: Phase types and lattice parameters for compound **2** (n = 8) as a function of temperature (°C).

Temperature (°C)	Phase type	Lattice parameters (nm)
r.t. to 100	crystal	-
Phase transition		
100		5.04
120		5.01
140		4.88
160		4.78
180		4.68
200		4.60
220	Liquid	-

Table S4: Phase types and lattice parameters for compound **3** (n = 10) as a function of temperature (°C).

Temperature (°C)	Phase Type	Lattice parameters (nm)
r.t. to 130	crystal	-
	Phase transition	
140	$Col_{rec}/p2$	*a* = 6.10 nm<br>*b* = 5.67 nm<br>© = 125.9°
150		*a* = 5.93 nm<br>*b* = 5.54 nm<br>γ = 126.0°
	Phase transition	
170	Cub/ $Ia\bar{3}d$	a_{cub} = 11.41 nm
180		a_{cub} = 11.27 nm
	Phase transition	
200	Lamellar	4.52

Table S5: Phase types and lattice parameters for compound **4** (n = 12) as a function of temperature (°C).

Temperature (°C)	Phase Type	Lattice parameters (nm)
r.t. to 110	crystal	-
	Phase transition	
120	$Col_{rec}/p2$	*a* = 6.15 nm, *b* = 6.02 nm,<br>γ = 126.2°
130		*a* = 6.35 nm, *b* = 5.94 nm,<br>γ = 127.1°
	Phase transition	
150	Cub/ $Ia\bar{3}d$	a_{cub} = 11.28 nm
160		a_{cub} = 11.06 nm
170		No spectra recorded
	Phase transition	
190	Lamellar	*d* = 4.40 nm
200		*d* = 4.37 nm

Table S6: Phase types and lattice parameters for compound **5** (n = 14) as a function of temperature (°C).

Temperature (°C)	Phase Type	Lattice Parameters (nm)
r.t. to 130	crystal	-
Phase transition		
130	$Col_{rec}/p2$	a = 6.41 nm, b = 6.32 nm, γ = 126.2°
150		a = 6.33 nm, b = 5.97 nm, γ = 127.3°
Phase transition		
160	Cub/ $Ia\bar{3}d$	a_{cub} = 11.48 nm
170		No spectra recorded
180		
185		
190		
Phase transition		
195	Lamellar	d = 4.42 nm
200	Liquid	-

Table S7: Phase types and lattice parameters for compound **6** (n = 16) as a function of temperature (°C).

Temperature (°C)	Phase Type	Lattice Parameters (nm)
r.t. to 172	crystal	-
Phase transition		
174	Cub/ $Ia\bar{3}d$	a_{cub} = 12.10 nm
190		a_{cub} = 11.73 nm
200	Phase transition	-
210	Lamellar	d = 4.67 nm

Table S8: Phase types and lattice parameters for compound **7** (n = 18) as a function of temperature (°C).

Temperature (°C)	Phase Type	Lattice parameters (nm)
r.t. to 130	crystal	-
Phase transition		
140	$Col_{rec}/p2$	a = 6.60 nm, b = 6.20 nm, γ = 127.9°
150		a = 6.50 nm, b = 6.01 nm, γ = 129.1°
Phase transition		
160	Cub/ $Ia\bar{3}d$	a_{cub} = 12.01 nm
170		No spectra recorded
180		
190		
Phase transition		
200	Lamellar	d = 4.58 nm

Detailed Synthesis of Materials

2.1 Characterization of Intermediates and Products: Starting materials and reagents were purchased from Aldrich. Chromatography solvents were purchased from SDS and Carlo Erba. Reactions were monitored by TLC using glass silica gel plates (Merck 60 F_{254}). The plates were developed using vaporisation with a solution of 10% H_2SO_4 in EtOH (v/v). Flash-chromatography separations were performed using Merck Gerudan silica gel Si 60 (40-63mm). NMR spectra were recorded on Bruker AC or DRX spectrometers at 75.47 MHz (100.61 MHz, or 125.77 MHz) for ^{13}C NMR and 300.13 MHz (or 400.13 MHz, or 500.13 MHz) for ^{1}H NMR. Chemical shifts (d) are given in parts per million (ppm) and were measured relative to the signal of tetramethylsilane (d = 0). Mass spectra were recorded by the Centre de Spectrométrie de Masse of the Université Claude Bernard (Villeurbanne) using electrospray (ESI) technique. Microanalyses were performed by the Service Central d'Analyse of CNRS. Optical rotations were measured at 20 °C with a Perkin Elmer 241 polarimeter at 589 nm (sodium D line) and concentrations (c) are reported in g/100 mL. For procedures and data for compounds **9** to **12**, see refs [26].

Spectroscopic details (^{1}HNMR and ^{13}CNMR spectra) for compounds **1** (Figures **S4** and **S5**), **2** (Figures **S6** and **S7**), **3** (Figures **S8** and **S9**), **5** (Figures **S10** and **S11**), **6** (Figures **S12** and **S13**), and **7** (Figures **S14** and **S15**) respectively are given at the end of the Supplementary Information, along with verification of the position of carbamoyl group at *O*-2 in compound **6** (see labelled structure **S16**) by 2D NMR (Figures **S17** to **S18**).

2.2 Details of the Target Intermediates and Products: The structures and compound numbers for the various target materials are given in Figure **19** below.

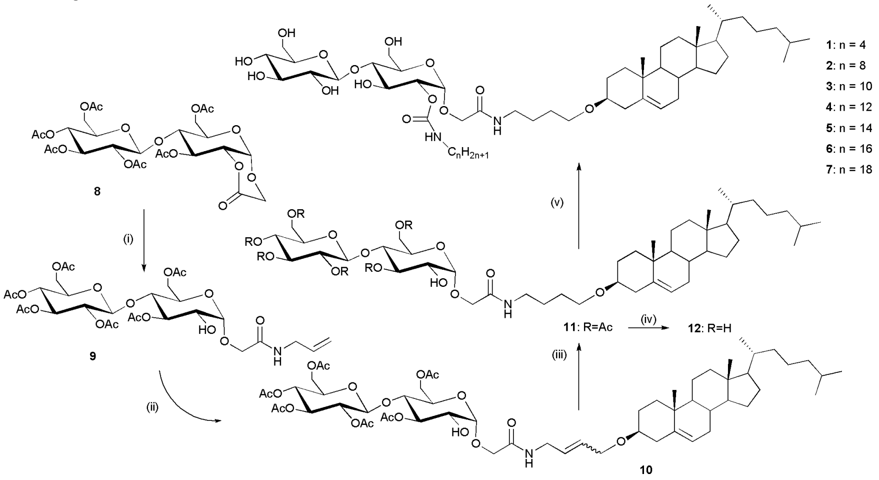

Figure S19: Intermediates and final products

2.3 Specific Synthetic Methods

General procedure for the synthesis of compounds 1-7

To a solution of alcohol **11** (0.09 mmol) in CH_2Cl_2 (2 mL), the appropriate alkyl isocyanate (0.27 mmol) and DBU (40 mL, 0.27 mmol) was added under nitrogen. The mixture was stirred for 15h at room temperature, and the white solid formed during the reaction was filtrated off and the solvent was removed by evaporation. The residue obtained was dissolved in a $MeOH/Et_3N/H_2O$ (8/1/1, 10 mL) mixture and was further stirred at 40°C for 3h. The reaction mixture was then co-evaporated three times with water and the resulting white residue was subjected to silica gel chromatography using a CH_2Cl_2/MeOH/Acetone/H_2O (67:15:15:3) to give the corresponding desired urethanes **1-7** in a ca. 50 to 60 mg scale [26].

2.4 Analytical data for the Final Products

***N*-{4-[(3β)-cholest-5-en-3-yloxy]butyl}-2-({2-*O*-[(butylamino)carbonyl]-4-*O*-β-D-glucopyranosyl-α-D-glucopyranosyl}oxy)acetamide (1).**

Yield: 57 %. White solid; ^{1}HNMR δ_H (400 MHz; $CDCl_3$/MeOD=7/3; Me_4Si) 7.16 (1 H, t, *J* 5.6, N-H), 6.59 (1 H, t, *J* 5.3, N-H), 5.30-5.22 (1 H, m, H-6''), 4.90 (1 H, d, *J* 3.8, H-1), 4.52 (1 H, dd, *J* 3.8 and 10.1, H-2), 4.90 (1 H, d, *J* 8.1, H-1'), 4.05 (1 H, d, *J* 15.6, H-7a), 3.94-3.69 (5 H, m, H-3, H-6a, H-6'a, H-6b, H-7b), 3.66-3.56 (3 H, m, H-4, H-5, H-6'b), 3.42-3.38 (2 H, m, CH_2-12), 3.33-3.15 (6 H, m, H-2', H-3', H-4', H-5', CH_2-9), 3.13-2.95 (3 H, m, H-3'', CH_2-14), 2.28 (1 H, ddd, *J* 13.2, 4.5 and 2.0, H-4''a), 2.16-2.03 (1 H, m, H-4''b), 1.99-0.70 (49 H, m, H-Cholesterol, CH_2-10, CH_2-11, CH_2-15 to CH_3-17), 0.61 (3 H, s, CH_3-18''); ^{13}C NMR δ_C (100 MHz; $CDCl_3$/MeOD=7/3; Me_4Si) 169.6, 156.1, 140.2, 121.4, 102.8, 96.8, 79.0, 78.8, 76.3, 76.1, 73.0, 72.7, 70.9, 69.5, 69.5, 69.4, 67.2, 66.2, 60.8, 60.0, 56.4, 55.8, 49.9, 41.9, 40.3, 39.4, 39.1, 38.6, 38.4, 36.8, 36.4, 35.8, 35.4, 31.5, 31.4, 27.9, 27.8, 27.5, 26.8, 25.8, 23.8, 23.4, 22.1, 21.8, 20.6, 19.4, 18.7, 18.0; $[\alpha]_D^{20}$ +10 (*c* 0.5 in $CHCl_3$/MeOH (1/1)); *m/z* (HRMS) 961.5986 (M + Na^+, $C_{50}H_{86}N_2O_{14}$, requires 961.5974).

***N*-{4-[(3β)-cholest-5-en-3-yloxy]butyl}-2-({2-*O*-[(octylamino)carbonyl]-4-*O*-β-D-glucopyranosyl-α-D-glucopyranosyl}oxy)acetamide (2).**

Yield: 65 %. White solid (Found: C, 64.92; H, 9.50; N, 2.88. $C_{54}H_{94}O_{14}N_2$ requires C, 65.16; H, 9.52; N, 2.81); ^{1}HNMR δ_H (300 MHz; $CDCl_3$/MeOD=7/3; Me_4Si) 5.21 (1 H, m, H-6''), 4.85 (1 H, d, *J* 3.8, H-1), 4.45 (1 H, dd, *J* 3.8 and 9.9, H-2), 4.29 (1 H, d, *J* 7.7, H-1'), 3.96 (1 H, d, *J* 15.6, H-7a), 3.91-3.62 (5 H, m, H-3, H-6a, H-6'a, H-6b, H-7b), 3.64-3.49 (3 H, m, H-4, H-5, H-6'b), 3.34 (2 H, m, CH_2-12), 3.22-3.16 (3 H, m, H-3', H-4', H-5'), 3.15-3.06 (3 H, m, H-2' CH_2-9), 3.06-2.86 (3 H, m, H-3'', CH_2-14), 2.20 (1 H, m, H-4''a), 2.04 (1 H, m, H-4''b), 1.93-0.60 (57 H, m, H-Cholesterol, CH_2-10, CH_2-11, CH_2-15 to CH_2-20, CH_3-21), 0.52 (3 H, s, CH_3-18''); ^{13}C NMR δ_C (101MHz; $CDCl_3$/MeOD=7/3; Me_4Si) 169.56, 156.13, 140.34, 129.47, 121.60, 102.89, 96.93, 79.15, 78.79, 77.48, 77.16, 76.84, 76.38, 76.13, 72.97, 72.89, 70.94, 69.55, 67.30, 66.39, 60.93, 60.21, 56.54, 55.93, 49.99, 49.06, 48.85, 48.64, 48.42, 48.21, 48.00, 47.78, 46.25, 42.08, 40.84, 39.55, 39.27, 38.81, 38.61, 36.96, 36.60, 35.94, 35.56, 31.67, 31.59, 29.52, 29.41, 29.07, 29.02, 28.11, 27.97, 27.73, 27.04, 26.62, 26.01, 24.01, 23.55, 22.39, 22.14, 20.82, 19.00, 18.36, 13.67, 11.50; $[\alpha]_D^{20}$ +37 (*c* 0.7 in $CHCl_3$/MeOH (3/7)); *m/z* (ESI) 995.6781 (M+H^+,$C_{54}H_{94}N_2O_{14}$ requires 995.6705).

***N*-{4-[(3β)-cholest-5-en-3-yloxy]butyl}-2-({2-*O*-[(decylamino)carbonyl]-4-*O*-β-D-glucopyranosyl-α-D-glucopyranosyl}oxy)acetamide (3).**

Yield: 50 %. White solid; ^{1}HNMR δ_H (400 MHz; $CDCl_3$/MeOD=7/3; Me_4Si) 7.16 (1 H, t, N-H), 6.59 (1 H, t, N-H), 5.31-5.23 (1 H, m, H-6''), 4.91 (1 H, d, *J* 3.8, H-1), 4.53 (1 H, dd, *J* 3.8 and 10.0, H-2), 4.35 (1 H, d, *J* 3.8, H-1'), 4.05 (1 H, d, *J* 15.6, H-7a), 3.96-3.68 (5 H, m, H-3, H-6a, H-6'a, H-6b, H-7b), 3.67-3.55 (3 H, m, H-4, H-5, H-6'b), 3.47-3.38 (2 H, m, CH_2-12), 3.34-3.15 (6 H, m, H-2', H-3', H-4', H-5', CH_2-9), 3.13-2.95 (3 H, m, H-3'', CH_2-14), 2.33-2.21 (1 H, m, H-4''a), 2.16-2.02 (1 H, m, H-4''b), 2.01-0.70 (61 H, m, H-Cholesterol, CH_2-10, CH_2-11, CH_2-15 to CH_2-22, CH_3-23), 0.61 (3 H, s, CH_3-18''); ^{13}C NMR δ_C (100 MHz; $CDCl_3$/MeOD=7/3; Me_4Si) δ 169.5, 156.1, 140.1, 121.7, 102.8, 96.8, 798.9, 78.7, 76.3, 76.0, 73.0, 72.7, 70.8, 69.5, 69.3, 67.4, 66.1, 60.8, 59.9, 56.3, 55.7, 49.8, 41.8, 40.5, 39.3, 39.0, 38.6, 38.3, 36.7, 36.3, 35.7, 35.3, 31.5, 29.3, 29.1, 28.9, 27.9, 27.9, 27.7, 27.5, 26.8, 26.4, 25.8, 23.7, 23.3, 22.1, 21.9, 21.7, 20.6, 18.6, 18.6, 17.9, 13.2, 11.1; $[\alpha]_D^{20}$ +8 (*c* 0.4 in $CHCl_3$/MeOH (1/1)); *m/z* (HRMS) 1045.6922 (M + Na^+, $C_{56}H_{98}N_2O_{14}$ requires 1045.6917).

***N*-{4-[(3β)-cholest-5-en-3-yloxy]butyl}-2-({2-*O*-[(dodecylamino)carbonyl]-4-*O*-β-D-glucopyranosyl-α-D-glucopyranosyl}oxy)acetamide (4) Compound 4 [27]:**

***N*-{4-[(3β)-cholest-5-en-3-yloxy]butyl}-2-({2-*O*-[(tetradecylamino)carbonyl]-4-*O*-β-D-glucopyranosyl-α-D-glucopyranosyl}oxy)acetamide (5).**

Yield: 65 %. White solid; ^{1}HNMR δ_H (300 MHz; $CDCl_3$/MeOD=7/3; Me_4Si) 5.27 (1 H, m, H-6''), 4.92 (1 H, d, *J* 3.6, H-1), 4.53 (1 H, dd, *J* 3.6 and 10.1, H-2), 4.38 (1 H, d, *J* 7.8, H-1'), 4.05 (1 H, d, *J* 15.7, H-7a), 3.96-3.69 (5 H, m, H-3, H-6a, H-6'a, H-6b, H-7b), 3.70-3.54 (3 H, m, H-4, H-5, H-6'b), 3.50 (2 H, m, CH_2-12), 3.36-3.14(6 H, m, H-2', H-3', H-4', H-5', CH_2-9), 3.13-2.97 (3 H, m, H-3'', CH_2-14), 2.27 (1 H, m, H-4''a), 2.12 (1 H, m, H-4''b), 2.05-0.65 (69 H, m, H-Cholesterol, CH_2-10, CH_2-11, CH_2-15 to CH_2-26, CH_3-27), 0.61 (3H, s, CH_3-18''); ^{13}C NMR δ_C (101 MHz, $CDCl_3$/MeOD=7/3) 169.56, 156.13, 140.35, 121.62, 102.90, 96.96, 79.18, 78.78, 77.48, 77.35, 77.16, 76.84, 76.39, 76.14, 72.96, 70.95, 69.55, 67.32, 66.38, 60.94, 60.23, 56.57, 55.96, 50.01, 49.10, 48.88, 48.67, 48.46, 48.25, 48.03, 47.82, 42.10, 40.89, 39.57, 39.29, 38.83, 38.63, 36.99, 36.62, 35.96, 35.58, 31.70, 29.56, 29.51, 29.45, 29.17, 28.14, 27.99, 27.76, 27.07, 26.69, 26.04, 24.03, 23.58, 22.43, 22.16, 20.85, 19.04, 18.38, 13.71, 11.53; $[\alpha]_D^{20}$ +31 (*c* 1.0 in $CHCl_3$/MeOH (9/1)); *m/z* (ESI) 1079.7709 (M+H$^+$,$C_{60}H_{106}N_2O_{14}$ requires 1079.7644).

***N*-{4-[(3β)-cholest-5-en-3-yloxy]butyl}-2-({2-*O*-[(hexadecylamino)carbonyl]-4-*O*-β-D-glucopyranosyl-α-D-glucopyranosyl}oxy)acetamide (6).**

Yield: 64 %.White solid (Found: C, 66.38; H, 9.96; N, 2.90. $C_{62}H_{110}O_{14}N_2$•H_2O requires C, 66,16; H, 10.03; N, 2.49); ^{1}HNMR δ_H (400 MHz; $CDCl_3$/MeOD=7/3; Me_4Si) 5.27 (1 H, m, H-6''), 4.92 (1 H, d, *J* 3.7, H-1), 4.52 (1 H, dd, *J* 3.7 and 10.0, H-2), 4.38 (1 H, d, *J* 7.8, H-1'), 4.04 (1 H, d, *J 15.6*, H-7a), 3.97-3.70 (5 H, m, H-3, H-6a, H-6'a, H-6b, H-7b), 3.63 (3 H, m, H-4, H-5, H-6'b), 3.40 (2 H, m, CH_2-12), 3.36-3.17 (6 H, m, H-2', H-3', H-4', H-5', CH_2-9), 3.14-2.96 (3 H, m, H-3'', CH_2-14), 2.28 (1 H, m, H-4''a), 2.11 (1 H, m, H-4''b), 1.67-0.65 (73 H, m, H-Cholesterol, CH_2-10, CH_2-11, CH_2-15 to CH_2-28, CH_3-29), 0.61 (3 H, s, CH_3-18''); ^{13}C NMR δ_C (101 MHz; $CDCl_3$/MeOD=7/3; Me_4Si) 169.51, 156.11, 140.50, 121.72, 102.95, 97.03, 79.27, 78.77, 77.48, 77.44, 77.34, 77.16, 77.12, 76.84, 76.79, 76.41, 76.19, 73.02, 72.90, 70.99, 69.67, 69.54, 67.42, 66.55, 61.04, 60.49, 56.67, 56.07, 50.11, 49.48, 49.26, 49.05, 48.84, 48.62, 48.41, 48.20, 45.95, 42.22, 41.03, 39.68, 39.41, 38.96, 38.72, 37.10, 36.74, 36.08, 35.69, 31.83, 31.80, 30.68, 29.69, 29.63, 29.57, 29.30, 29.27, 28.27, 28.12, 27.89, 27.21, 26.81, 26.17, 24.17, 23.72, 22.62, 22.57, 22.37, 20.97, 19.21, 18.56, 13.92, 11.70; $[\alpha]_D^{20}$ +29 (*c* 2.8 in $CHCl_3$/MeOH (7/3)); *m/z* (ESI) 1107.8027 (M+H$^+$,$C_{62}H_{110}N_2O_{14}$ requires 1107.7957).

***N*-{4-[(3β)-cholest-5-en-3-yloxy]butyl}-2-({2-*O*-[(octadecylamino)carbonyl]-4-*O*-β-D-glucopyranosyl-α-D-glucopyranosyl}oxy)acetamide (7).**

Yield: 58 %. White solid; ^{1}HNMR δ_H (400 MHz; $CDCl_3$/MeOD=7/3; Me_4Si) 5.26 (1 H, m, H-6''), 4.91 (1 H, d, *J* 3.5, H-1), 4.51 (1 H, dd, *J* 3.5 and 10.0, H-2), 4.37 (1 H, d, *J* 7.8, H-1'), 4.03 (1 H, d, *J 15.6*, H-7a), 3.89-3.66 (5 H, m, H-3, H-6a, H-6'a, H-6b, H-7b), 3.66-3.52 (3 H, m, H-4, H-5, H-6'b), 3.50 (2 H, m, CH_2-12), 3.37-3.26 (6 H, m, H-2', H-3', H-4', H-5', CH_2-9), 3.09-3.97 (3 H, m, H-3'', CH_2-12), 2.31 (1 H, m, H-4''a), 2.19 (1 H, m, H-4''b), 2.03-0.70 (77 H, m, H-Cholesterol, CH_2-10, CH_2-11, CH_2-15 to CH_2-30, CH_3-31), 0.60 (3 H, s, CH_3-18''); ^{13}C NMR δ_C (101 MHz; $CDCl_3$/MeOD=7/3; Me_4Si) 169.33, 156.05, 140.38, 121.89, 102.92, 97.14, 79.14, 78.66, 77.42, 77.31, 77.11, 76.79, 76.39, 76.17, 72.85, 71.00, 69.70, 69.50, 67.81, 66.73, 61.04, 60.55, 56.69, 56.09, 53.39, 50.11, 49.86, 49.64, 49.43, 49.21, 49.00, 48.79, 48.57, 48.36, 42.24, 41.07, 40.17, 39.69, 39.44, 38.92, 37.08, 36.76, 36.11, 35.72, 31.85, 31.81, 30.76, 29.67, 29.65, 29.62, 29.59, 29.57, 29.33, 29.29, 29.28, 28.26, 28.14, 27.92, 26.83, 24.20, 23.75, 22.68, 22.60, 22.42, 20.99, 20.88, 19.23, 18.60, 13.98, 11.74; $[\alpha]_D^{20}$ +28 (*c* 1.0 in $CHCl_3$/MeOH (8/2)); *m/z* (HRMS) 1135.8386 (M +H$^+$, $C_{64}H_{114}O_{14}N_2$ requires 1134.8270).

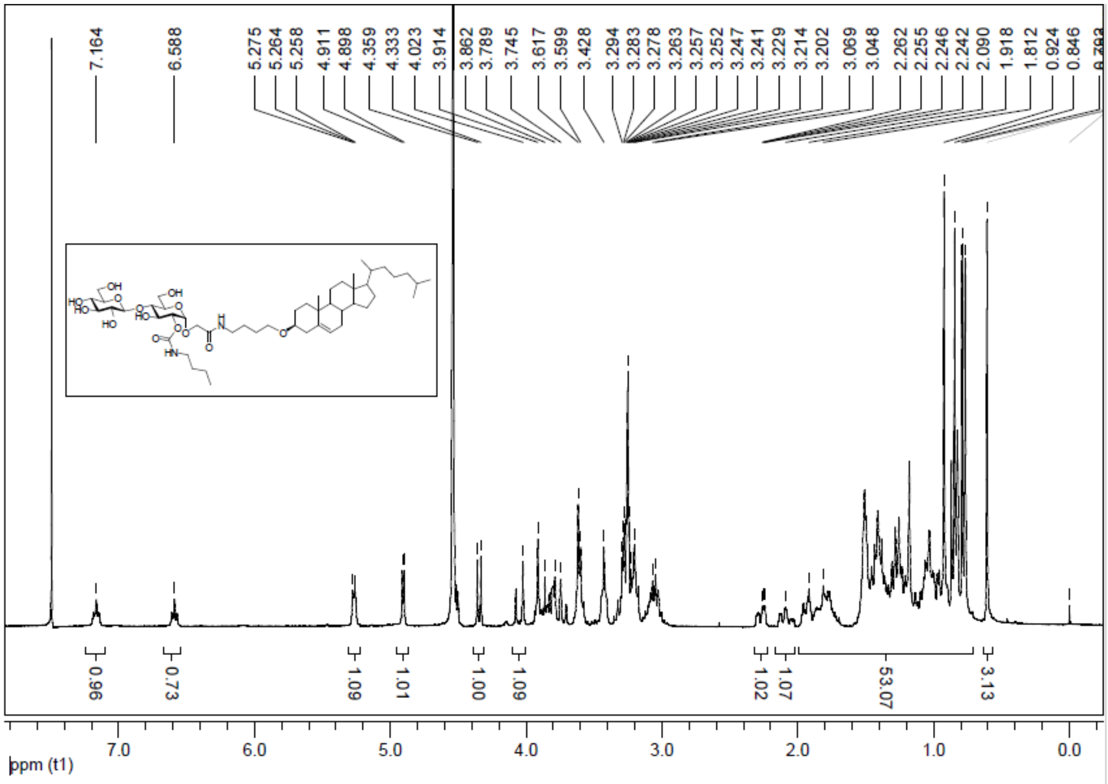

Figure S4: ^{1}HNMR spectra of compound **1**

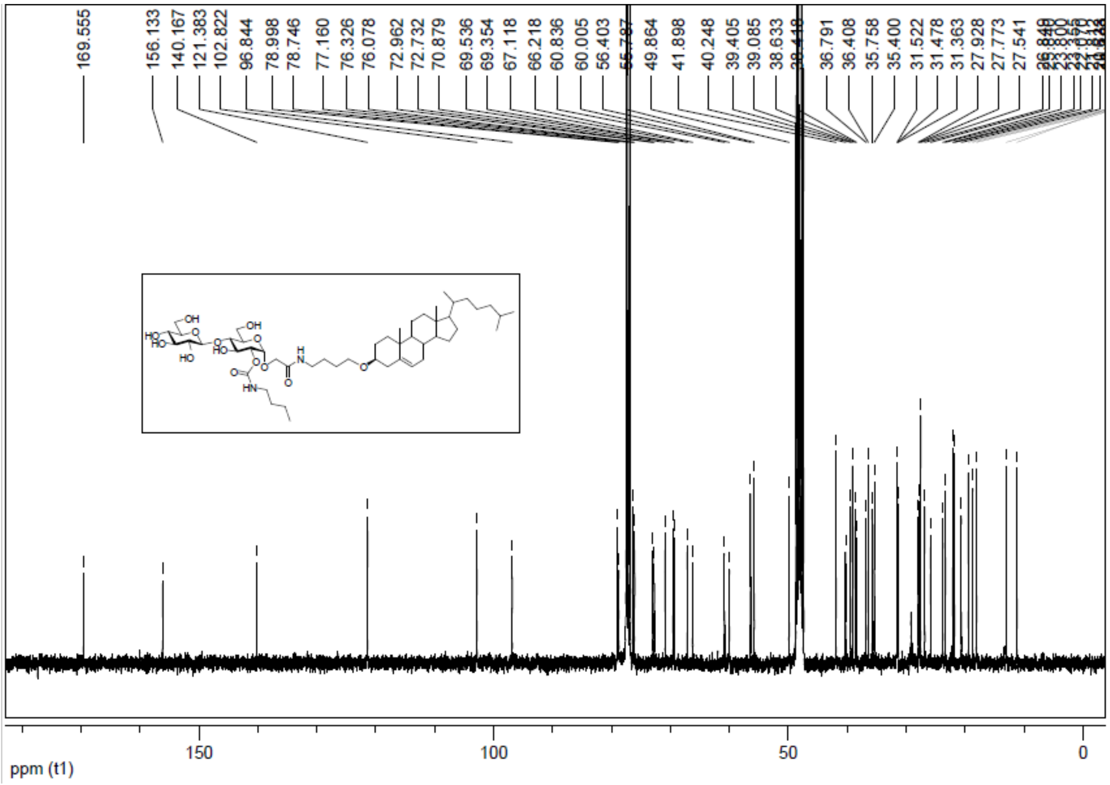

Figure S5: ^{13}CNMR spectra of compound **1**

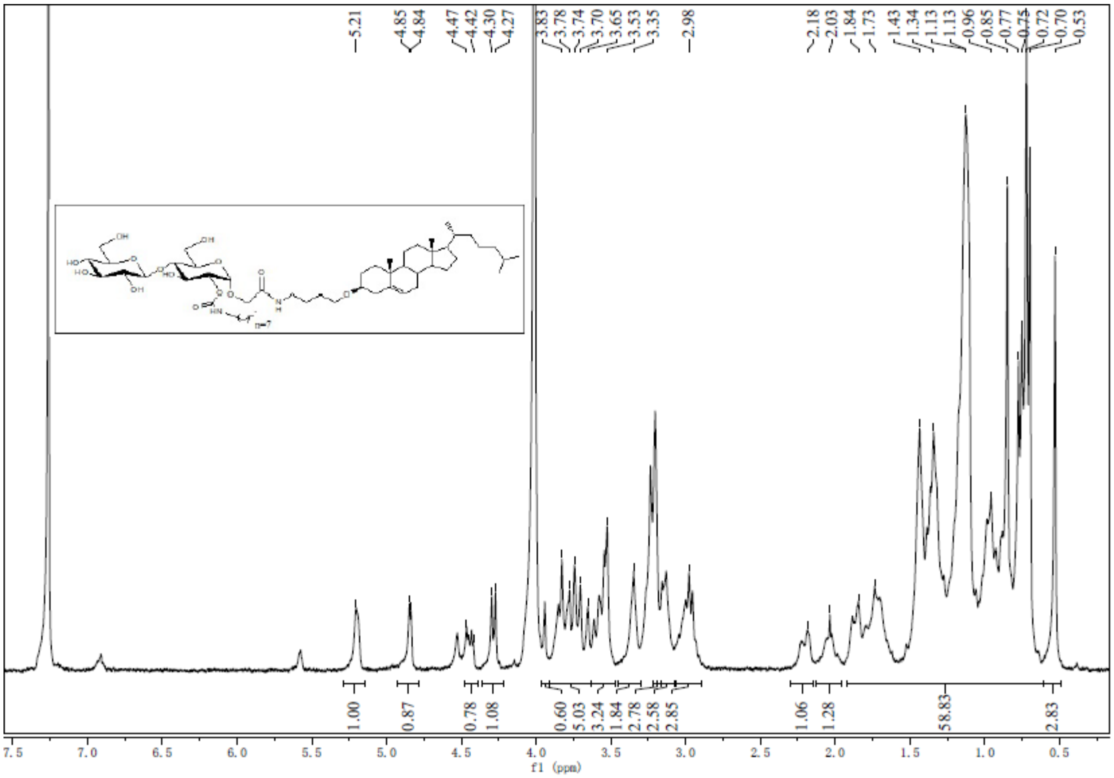

Figure S6: ^{1}HNMR spectra of compound **2**.

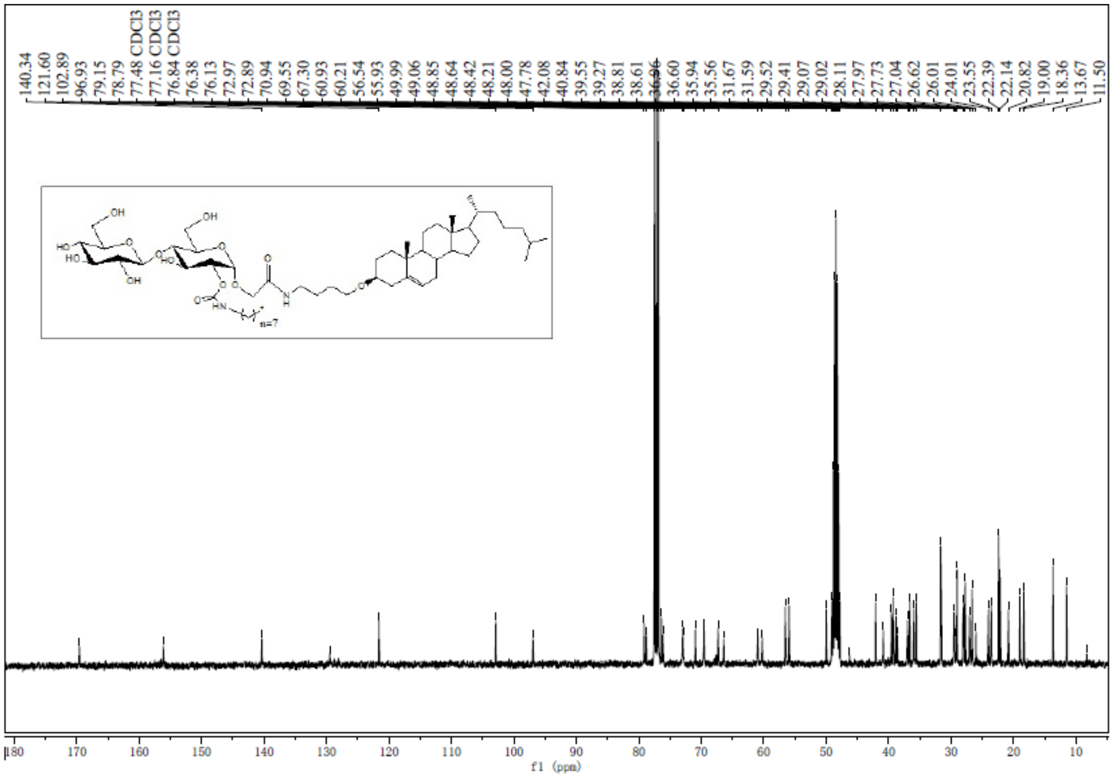

Figure S7: ^{13}CNMR spectra of compound **2**.

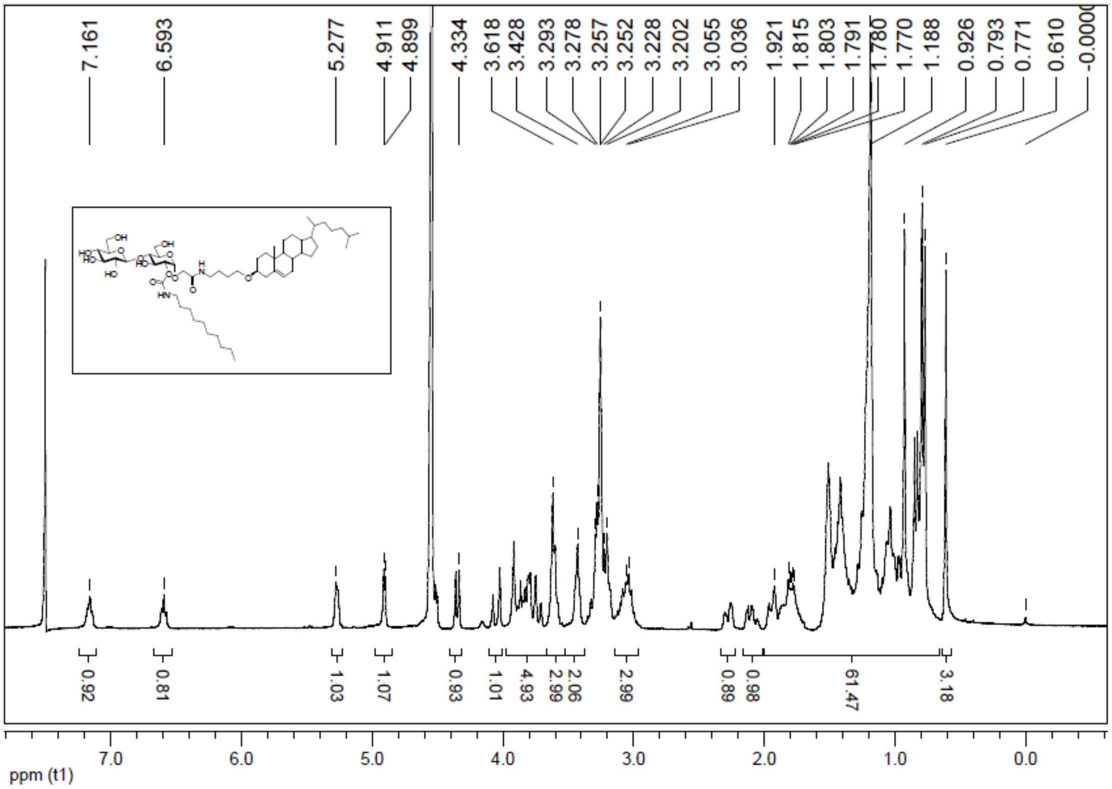

Figure S8: ^{1}HNMR spectra of compound **3**.

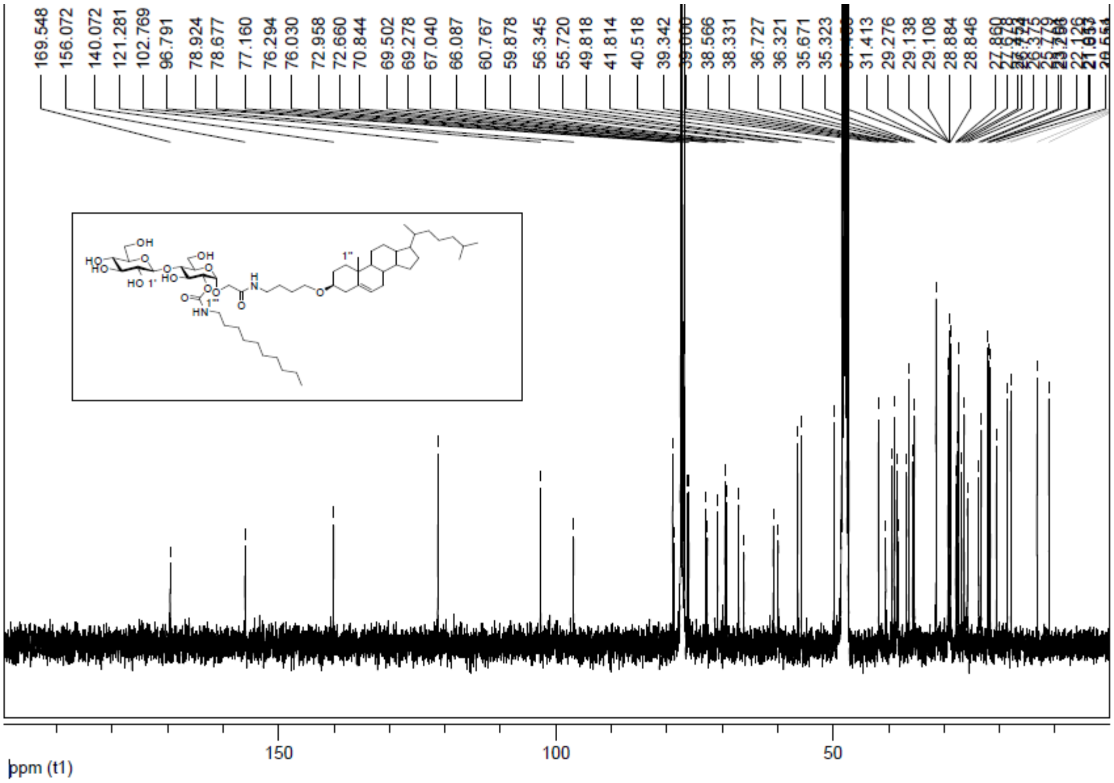

Figure S9: ^{13}CNMR spectra of compound **3**.

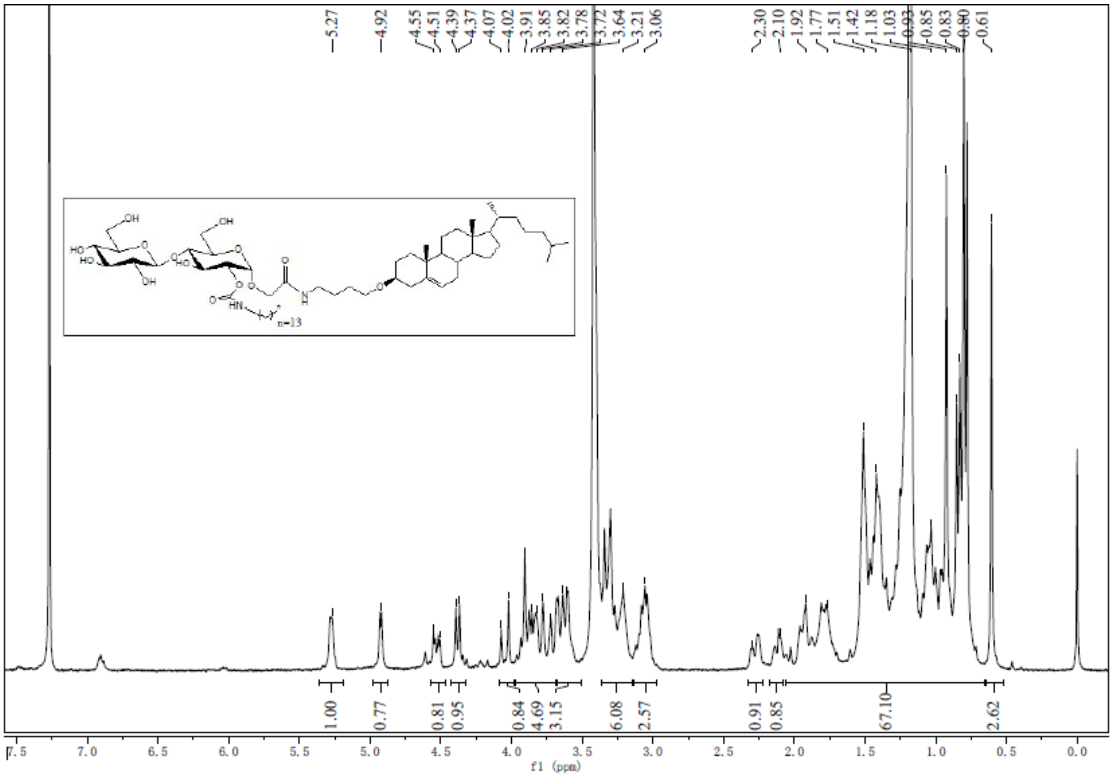

Figure S10: ^{1}HNMR spectra of compound **5**.

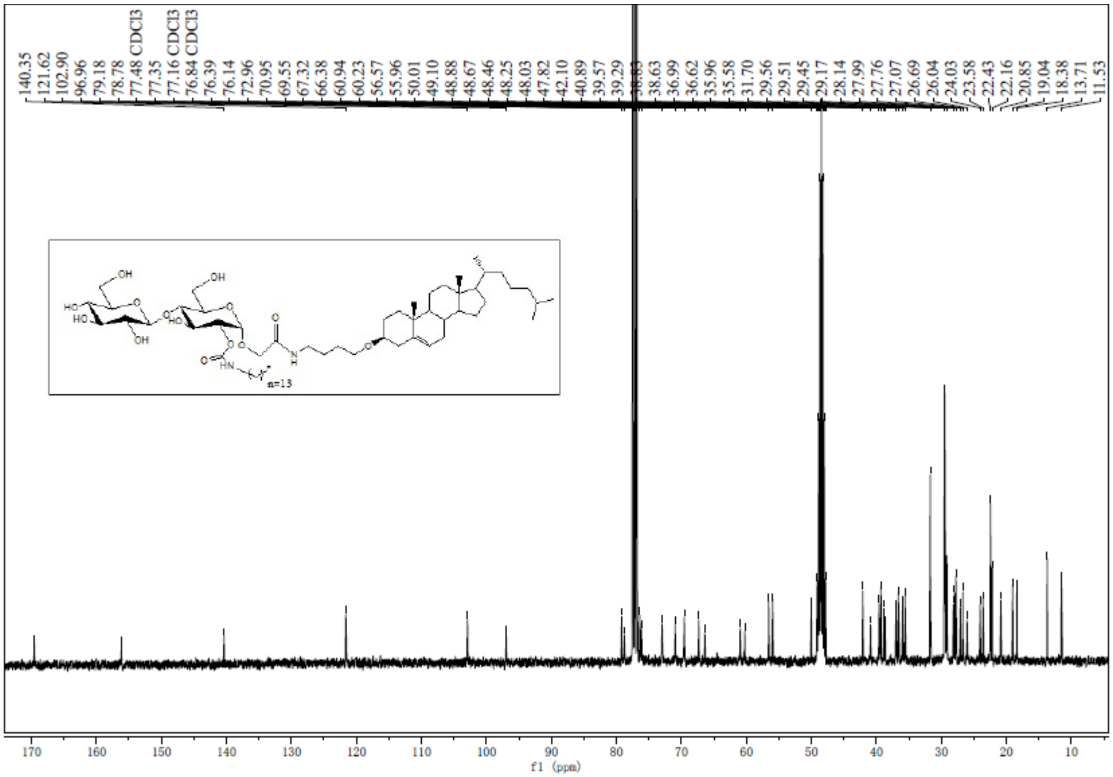

Figure S11: ^{13}CNMR spectra of compound **5**.

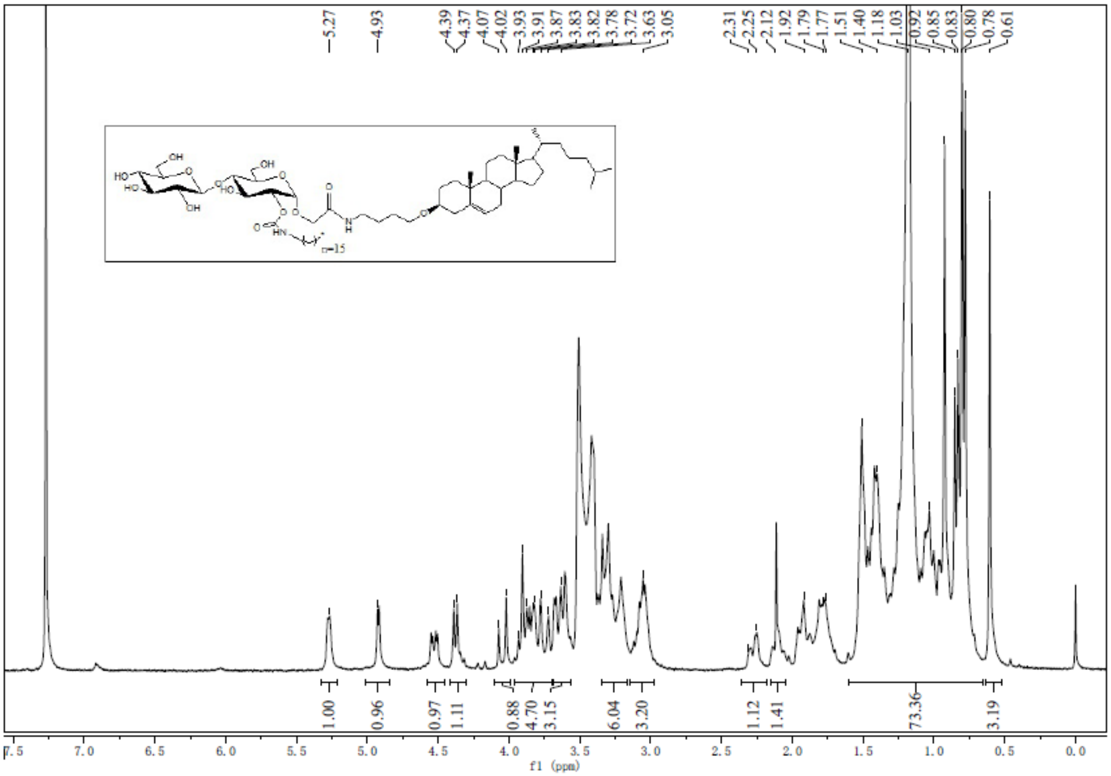

Figure S12: ^{1}HNMR spectra of compound **6**.

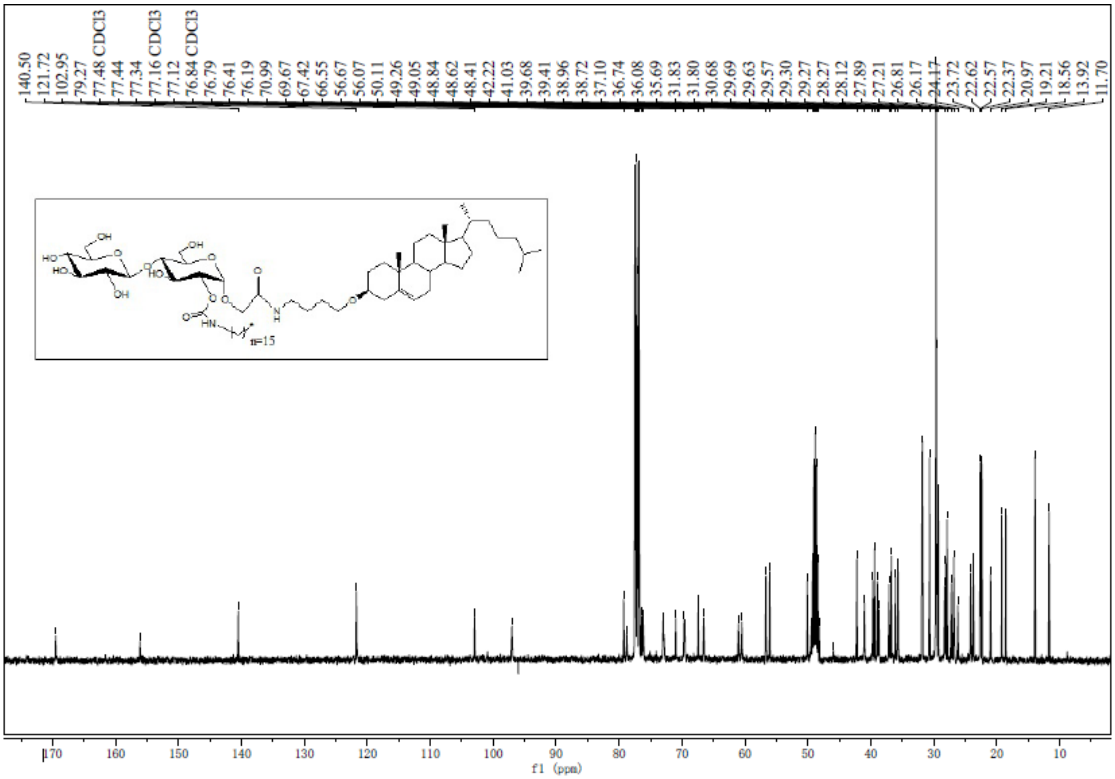

Figure S13: ^{13}CNMR spectra of compound **6**.

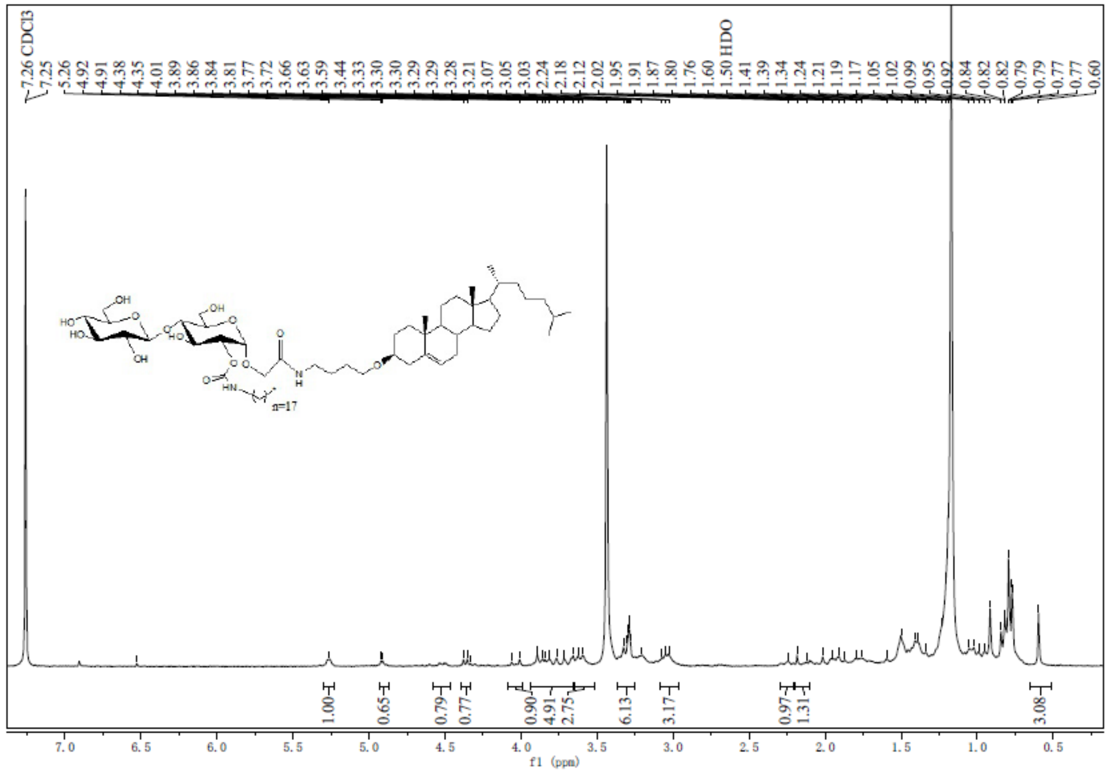

Figure S14: ^{1}HNMR spectra of compound **7**.

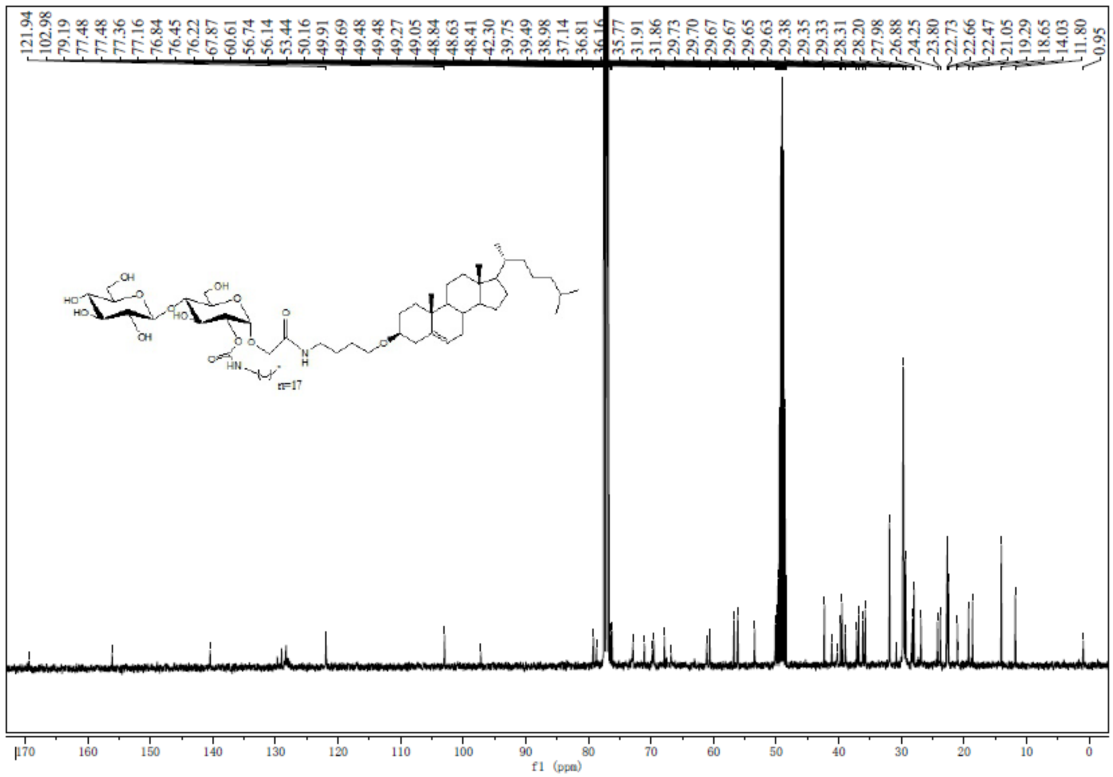

Figure S15: ^{13}CNMR spectra of compound **7.**

2.5 Verification of the position of carbamoyl group at O-2 in compound 6 by 2D NMR.

The position of the carbamoyl chain at O-2 in the final compounds is unambiguous as it arises from the reaction of the 2-monohydroxy precursor **11** with all other positions protected**,** itself arising from the opening of the lactone. This can be also fully confirmed by checking 2D NMR Heteronuclear Multiple Bond Connectivity (HMBC) correlations between H2 and the carbamoyl group. For this, a first 2D HSQC experiment (Figure S14) allowed to identify H2 in **6**, showing the proton H2 at 4.52 ppm correlated with the carbon C2 at δ73.02ppm. After H2 was indentified, the HMBC spectrum (Figure S15), exhibit correlations between the protons of H2 and H14 and the C13 carbon signal at δ156.11 ppm, that is the carbonyl group in the fatty chain.

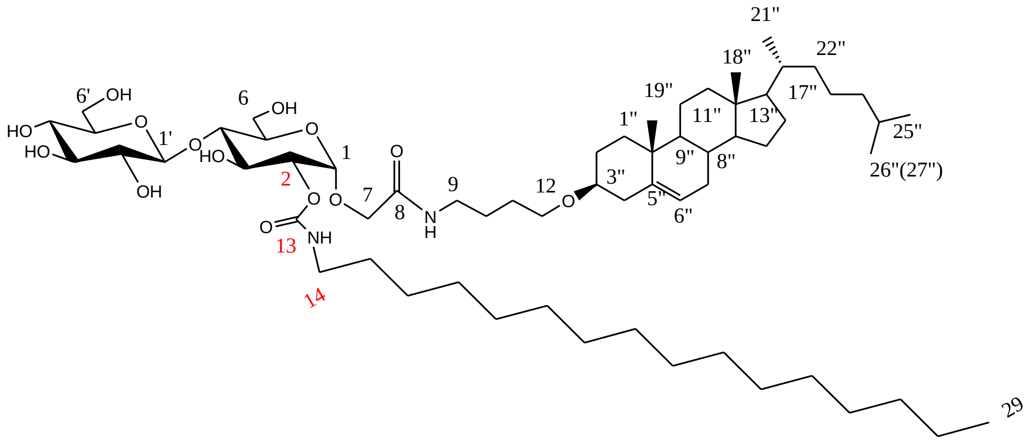

Figure S16: Structure of compound **6.**

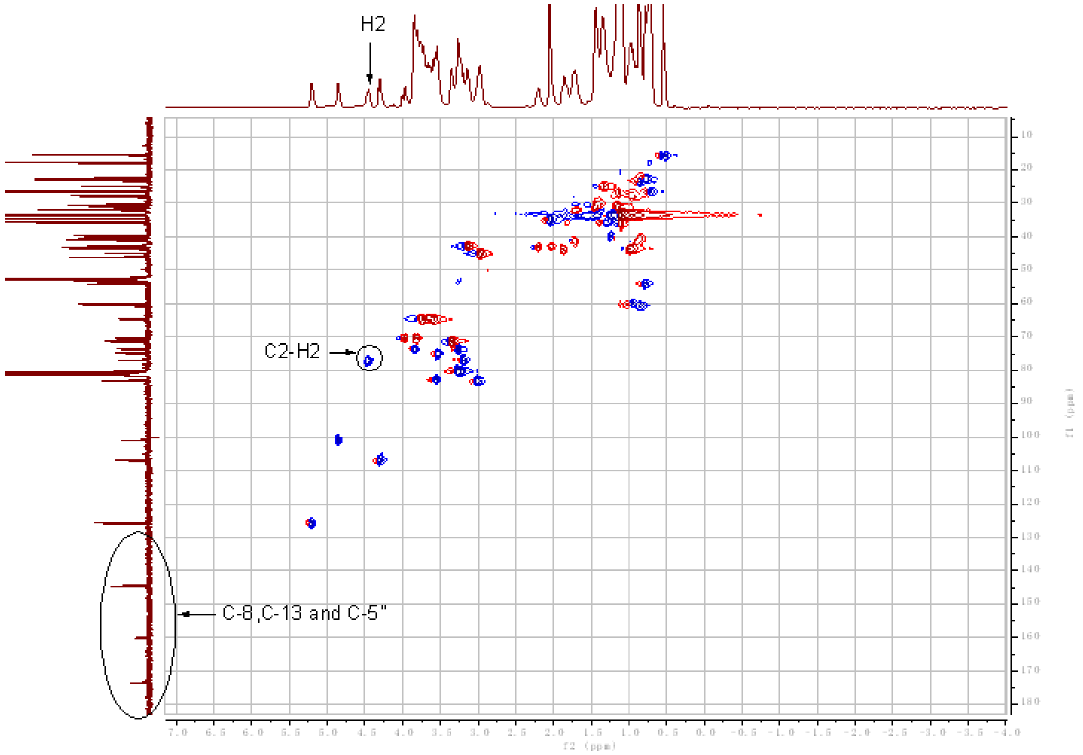

Figure S17: HSQC spectra of compound **6** in $CDCl_3$/MeOD=7/3.

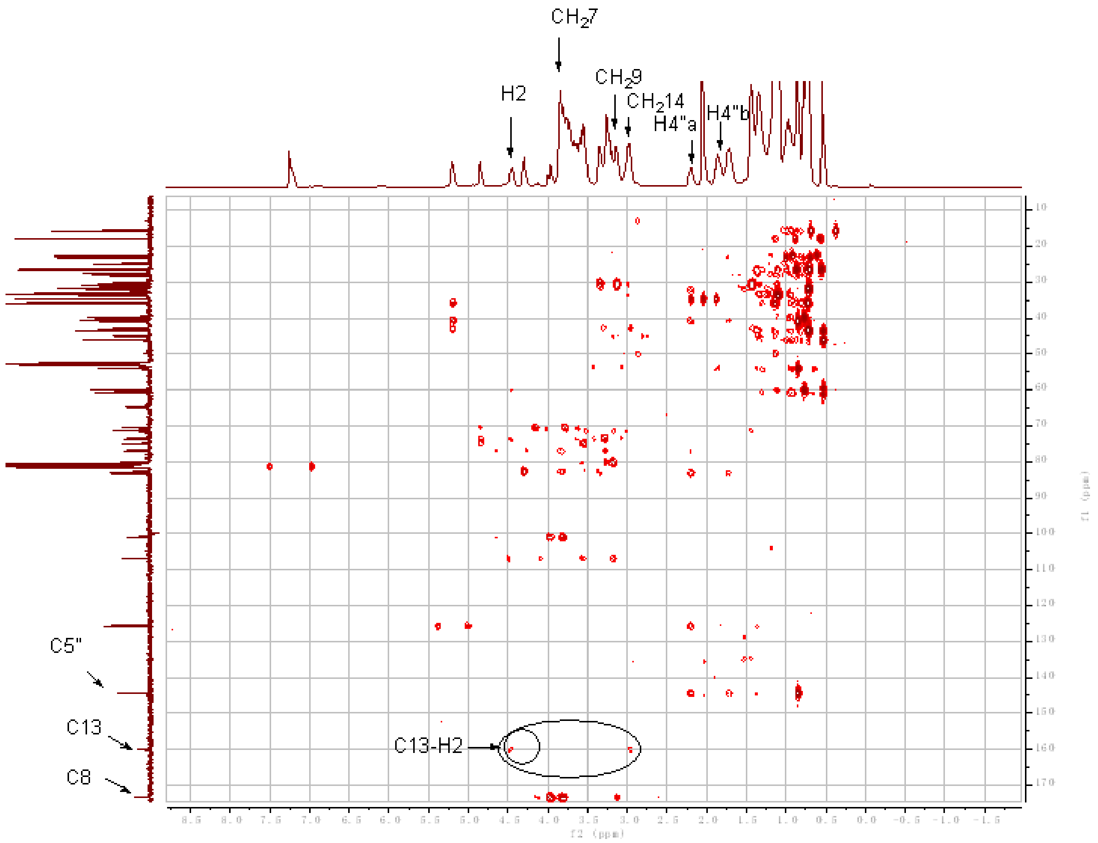

Figure S18: HMBC spectra of compound **6** in $CDCl_3$/MeOD=7/3.

References:

[1] M.J.S. Dewar, E.G. Zoebisch, E.F. Healy, J. Am. Chem. Soc., 1985, **107**, 3902–3909.

[2] A.D. Becke, *J. Chem. Phys.,* 1993, **98**, 5648–52.

[3] C.T. Lee, W.T. Yang, R.G. Parr, *Phys. Rev. B: Condens. Matter Mater. Phys.*, 1988, **37**, 785–789

[4] Gaussian 16, Revision A.03, M.J. Frisch, G.W. Trucks, H.B. Schlegel, G E. Scuseria, M.A. Robb, J.R. Cheeseman, G. Scalmani, V. Barone, G.A. Petersson, H. Nakatsuji, X. Li, M. Caricato, A.V. Marenich, J. Bloino, B.G. Janesko, R. Gomperts, B. Mennucci, H.P. Hratchian, J.V. Ortiz, A.F. Izmaylov, J.L. Sonnenberg, D. Williams-Young, F. Ding, F. Lipparini, F. Egidi, J. Goings, B. Peng, A. Petrone, T. Henderson, D. Ranasinghe, V.G. Zakrzewski, J. Gao, N. Rega, G. Zheng, W. Liang, M. Hada, M. Ehara, K. Toyota, R. Fukuda, J. Hasegawa, M. Ishida, T. Nakajima, Y. Honda, O. Kitao, H. Nakai, T. Vreven, K. Throssell, J.A. Montgomery, Jr., J.E. Peralta, F. Ogliaro, M.J. Bearpark, J.J. Heyd, E.N. Brothers, K. N. Kudin, V.N. Staroverov, T.A. Keith, R. Kobayashi, J. Normand, K. Raghavachari, A.P. Rendell, J.C. Burant, S.S. Iyengar, J. Tomasi, M. Cossi, J.M. Millam, M. Klene, C. Adamo, R. Cammi, J.W. Ochterski, R.L. Martin, K. Morokuma, O. Farkas, J.B. Foresman, D.J. Fox, Gaussian, Inc., Wallingford CT; 2016.

[5] D.A. Case, I.Y. Ben-Shalom, S.R. Brozell, D.S. Cerutti, T.E. Cheatham, III, V.W.D. Cruzeiro, T.A. Darden, R.E. Duke, D. Ghoreishi, G. Giambasu, T. Giese, M.K. Gilson, H. Gohlke, A.W. Goetz, D. Greene, R Harris, N. Homeyer, Y. Huang, S. Izadi, A. Kovalenko, R. Krasny, T. Kurtzman, T.S. Lee, S. LeGrand, P. Li, C. Lin, J. Liu, T. Luchko, R. Luo, V. Man, D.J. Mermelstein, K.M. Merz, Y. Miao, G. Monard, C. Nguyen, H. Nguyen, A. Onufriev, F. Pan, R. Qi, D.R. Roe, A. Roitberg, C. Sagui, S. Schott-Verdugo, J. Shen, C.L. Simmerling, J. Smith, J. Swails, R.C. Walker, J. Wang, H. Wei, L. Wilson, R.M. Wolf, X. Wu, L. Xiao, Y. Xiong, D.M. York, P.A. Kollman, AMBER 2019, University of California, San Francisco; 2019.

[6] C.I. Bayly, P. Cieplak, W. Cornell, P.A. Kollman, *J. Phys. Chem.*, 1993, **97**, 10269–10280.

[7] J. Wang, R.M. Wolf, J.W. Caldwell, P.A. Kollman, D.A. Case, *J. Comput. Chem.*, 2004, **25**, 1157–1174.

[8] A.W. Sousa da Silva, W.F. Vranken, *BMC Res. Notes*, 2012, **5**, 367.

[9] M.J. Abraham, T. Murtola, R. Schulz, S. Páll, J.C. Smith, B. Hess, E. Lindahl, *SoftwareX*, 2015, **1**–**2**, 19–25.

[10] S. Páll, M.J. Abraham, C. Kutzner, B. Hess, E. Lindahl, in Solving Software Challenges for Exascale: International Conference on Exascale Applications and Software, EASC 2014, Stockholm, Sweden, April 2-3, 2014, Revised Selected Papers, ed. S. Markidis and E. Laure, Springer International Publishing, Cham, 2015, pp. 3–27.

[11] S. Pronk, S. Páll, R. Schulz, P. Larsson, P. Bjelkmar, R. Apostolov, M.R. Shirts, J.C. Smith, P.M. Kasson, D. van der Spoel, B. Hess, E. Lindahl, *Bioinformatics*, 2013, **29**, 845–854.

[12] B. Hess, C. Kutzner, D. van der Spoel, E. Lindahl, *J. Chem. Theory Comput.*, 2008, **4**, 435–447.

[13] D. Van Der Spoel, E. Lindahl, B. Hess, G. Groenhof, A.E. Mark, H.J.C. Berendsen, *J. Comput. Chem.*, 2005, **26**, 1701–1718.

[14] E. Lindahl, B. Hess, D. van der Spoel, *Molecular Modeling Annual*, 2001, **7**, 306–317.

[15] H.J.C. Berendsen, D. Vanderspoel, R. Vandrunen, *Comput. Phys. Commun.*, 1995, **91**, 43–56.

[16] B. Hess, H. Bekker, H.J.C. Berendsen, J.G.E.M. Fraaije, *J. Comput. Chem.*, 1997, **18**, 1463–1472.

[17] S. Miyamoto, P.A. Kollman *J. Comp. Chem.* 1992, **13**, 952–962.

[18] U. Essmann, L. Perera, M.L. Berkowitz, T. Darden, H. Lee, L.G. Pedersen *J. Chem. Phys.* 1995, **103**, 8577–8592

[19] H.J.C. Berendsen, J.P.M. Postma, A. DiNola, J.R. Haak *J. Chem. Phys.*, 1984, **81**, 3684–3690.

[20] G. Bussi, D. Donadio, M. Parrinello, *J. Chem. Phys.*, 2007, **126**, 014101.

[21] S. Nosé, *Mol. Phys.*, 1984, **52**, 255–268.

[22] W.G. Hoover, *Phys. Rev. A: At., Mol., Opt. Phys.*, 1985, **31**, 1695–1697.

[23] M. Parrinello, A. Rahman, *J. Appl. Phys.*, 1981, **52**, 7182–7190.

[24] S. Nosé, M L. Klein, *Mol. Phys.*, 1983, **50**, 1055–1076.

[25] W. Humphrey, A. Dalke, K. Schulten J. Molec. Graphics, 1996, 14, 33–38.

[26] F. Ali-Rachedi, S.J. Cowling, S. Chambert, Y. Queneau, Chemistry Africa 2023, 6, 2419–2428.

[27] F. Ali Rachedi, S. Chambert, F. Ferkous, Y. Queneau, S.J. Cowling, J.W. Goodby, Chem. Commun., 2009, 6355-6357.
